# The C-terminal region of the oxidoreductase MIA40 stabilizes its cytosolic precursor during mitochondrial import

**DOI:** 10.1186/s12915-020-00824-1

**Published:** 2020-08-06

**Authors:** Lena Maria Murschall, Anne Gerhards, Thomas MacVicar, Esra Peker, Lidwina Hasberg, Stephan Wawra, Thomas Langer, Jan Riemer

**Affiliations:** 1 Institute for Biochemistry, Redox Biochemistry, University of Cologne, Zuelpicher Str. 47a, 50674 Cologne, Germany; 2 Department of Mitochondrial Proteostasis, Max Planck Institute for Biology of Ageing, 50931 Cologne, Germany; 3 Botanical Institute, Cluster of Excellence on Plant Sciences (CEPLAS), University of Cologne, 50674 Cologne, Germany; 4 Cologne Excellence Cluster on Cellular Stress Responses in Aging-Associated Diseases (CECAD), University of Cologne, 50931 Cologne, Germany

**Keywords:** MIA40, Negatively charged C-terminus, Mitochondrial precursor, Mitochondrial import, Disulfide relay, Proteasomal degradation

## Abstract

**Background:**

The mitochondrial intermembrane space (IMS) is home to proteins fulfilling numerous essential cellular processes, particularly in metabolism and mitochondrial function. All IMS proteins are nuclear encoded and synthesized in the cytosol and must therefore be correctly targeted and transported to the IMS, either through mitochondrial targeting sequences or conserved cysteines and the mitochondrial disulfide relay system. The mitochondrial oxidoreductase MIA40, which catalyzes disulfide formation in the IMS, is imported by the combined action of the protein AIFM1 and MIA40 itself. Here, we characterized the function of the conserved highly negatively charged C-terminal region of human MIA40.

**Results:**

We demonstrate that the C-terminal region is critical during posttranslational mitochondrial import of MIA40, but is dispensable for MIA40 redox function in vitro and in intact cells. The C-terminal negatively charged region of MIA40 slowed import into mitochondria, which occurred with a half-time as slow as 90 min. During this time, the MIA40 precursor persisted in the cytosol in an unfolded state, and the C-terminal negatively charged region served in protecting MIA40 from proteasomal degradation. This stabilizing property of the MIA40 C-terminal region could also be conferred to a different mitochondrial precursor protein, COX19.

**Conclusions:**

Our data suggest that the MIA40 precursor contains the stabilizing information to allow for postranslational import of sufficient amounts of MIA40 for full functionality of the essential disulfide relay. We thereby provide for the first time mechanistic insights into the determinants controlling cytosolic surveillance of IMS precursor proteins.

## Background

Proteins of the mitochondrial intermembrane space (IMS) fulfill numerous critical functions, including enzymatic functions in metabolic pathways such as pyrimidine, lipid and heme biosynthesis, functions in the maintenance of mitochondrial morphology and dynamics, in assembly of the respiratory chain, and in maintenance of the mitochondrial proteome [1–5]. All IMS proteins are nuclear encoded and synthesized at cytosolic ribosomes. For their import, some IMS proteins rely on so-called bipartite targeting sequences, which guide them in a TIMM23-dependent manner into the IMS. However, many proteins lack mitochondrial targeting sequences and instead rely on conserved cysteines and the mitochondrial disulfide relay system for their IMS import [6–9]. Most often, the conserved cysteines are arranged in twin-CX_n_C motifs, which consist of four cysteines positioned in opposing antiparallel α-helices [10, 11]. In the mature proteins, these cysteines form structural disulfide bonds. For recognition by the import machinery, substrates additionally contain the so-called intermembrane space targeting sequences (ITS, also called mitochondrial intermembrane space sorting signal, MISS) close to one of the cysteines [12, 13]. Many substrates of the mitochondrial disulfide relay are imported in a comparatively slow posttranslational manner, compared to MTS-containing proteins, possibly due to the tight link of oxidative protein folding and outer membrane translocation [14, 15]. Consequently, import is monitored by cytosolic quality control systems ensuring efficient import and avoiding the accumulation of precursors in the cytosol or clogging mitochondrial import channels [15–19]. The molecular determinants controlling this cytosolic surveillance are however only poorly understood and do not explain preferential targeting of some precursors but not others.

**Fig. 1.**
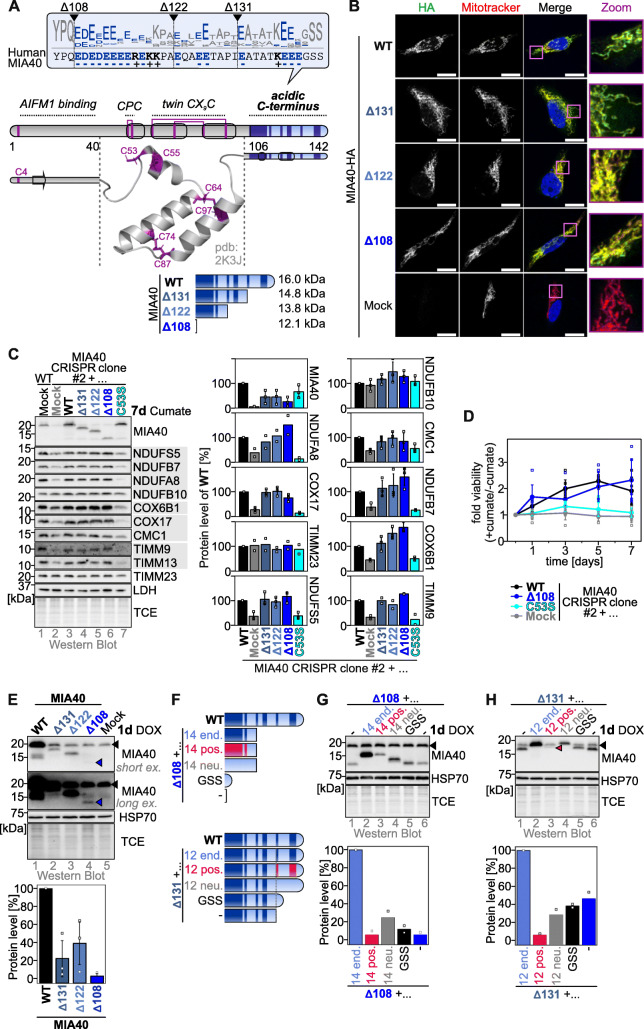
Truncation of the conserved negatively charged C-terminal region of human MIA40 decreases its cellular levels. **a** Conservation of the C-terminal domain of human MIA40. Human MIA40 consists of three parts, an N-terminal domain important for interaction with AIFM1 and MIA40 import, a core domain important for interaction with its substrates and their oxidation, and a negatively charged C-terminal region (indicated in light blue) with unknown function. The negative charges in the C-terminal region are clustered into three clusters (indicated in dark blue), and corresponding truncation mutants were employed for further studies. The logo plot was derived from alignments of MIA40 from 86 species. **b** Localization of HA-tagged MIA40 variants. HEK293 cells stably expressing the indicated MIA40-HA variants were incubated for 24 h with 1 μg ml^−1^ doxycycline to induce expression. Cells were fixed, permeabilized, and stained using a primary antibody against the HA epitope (HA, green) and Mitotracker (red). Nuclei were stained with DAPI (blue). Cells were analyzed by fluorescence microscopy. All MIA40 variants localize to mitochondria. Scale bar, 15 μm. *N* = 11–15 cells, 2 biological replicates. **c** Complementation of MIA40 CRISPR-Cas9 clone with truncated MIA40 variants and test of MIA40 substrate levels. Cell lines were stably transfected with inducible plasmids harboring the indicated variants of MIA40 or with the empty vector (Mock). Expression of MIA40 variants was induced for 7 days with 30 μg ml^−1^ cumate in glucose-containing medium. Cells were lysed, and the levels of the indicated proteins were analyzed by SDS-PAGE and immunoblotting (gray background: MIA40 substrates). Expression of MIA40^WT^ and all truncation variants, but not MIA40^C53S^, complemented the loss of endogenous MIA40. Quantification using Image Lab. Data from 2 to 3 experiments were combined and standard deviations are presented if *n* > 2. **d** Complementation of MIA40 CRISPR-Cas9 clone with truncated MIA40 variants and test of proliferation. Cell lines were stably transfected with inducible plasmids harboring the indicated variants of MIA40 or with the empty vector (Mock). Expression of MIA40 variants was induced with 30 μg ml^−1^ cumate in galactose-containing medium. Cells were analyzed by PrestoBlue cell viability reagent at the indicated times. Fold viability is presented as the viability data of induced cells divided by the data of non-induced cells of the same cell line. MIA40^C53S^ cannot rescue the growth of cells depleted of MIA40, while MIA40^WT^ and MIA40^Δ108^ can. Data from 6 experiments were combined and standard deviations are presented. **e** Steady-state levels of MIA40 variants. HEK293 cells stably and inducibly expressing different MIA40 variants were lysed 24 h after induction of MIA40 expression (1 μg ml^−1^ doxycycline, DOX), and then analyzed by immunoblotting. Truncated variants of MIA40 are present at strongly decreased levels compared to MIA40^WT^(black arrowhead, endogenous MIA40, blue arrowhead, MIA40^Δ108^). Quantification using Image Lab. Data from 3 experiments were combined and standard deviations are presented. **f**–**h** Steady-state levels of MIA40 variants. As **e**, except that different C-terminal extensions were fused to MIA40^Δ108^ (**f**, **g**) and MIA40^Δ131^ (**f**, **h**) and expressed stably, inducibly in HEK293 cells (induction with 1 μg ml^−1^ doxycycline, DOX for 24 h). Either the missing endogenous amino acid residues were added back (light blue bar), or we exchanged the negative amino acid residues in these add-back parts with positive (red bar, pos.) or neutral (gray bar, neu.) amino acid residues. Alternatively, we only added the most C-terminal amino acid residues of wild type MIA40, “GSS” (black bar). Only add-back of the endogenous parts containing the negative charges restored MIA40 levels. Quantification using Image Lab. Data from 2 experiments were combined. Black arrowhead, endogenous MIA40; red arrowhead, signal of MIA40^Δ131^ fused to the C-terminal region with negatively charged residues mutated to positively charged residues

The central component of the IMS disulfide relay machinery is the oxidoreductase MIA40 (or CHCHD4) that serves both as import receptor and oxidoreductase. Using a hydrophobic interaction interface, MIA40 interacts with incoming precursors in a non-covalent manner [13, 20–22]. Concomitantly, it employs a redox-active cysteine motif to oxidize and thereby import precursor proteins [15, 23]. Both, hydrophobic interface and redox-active cysteine motif are localized in the core domain of MIA40 which is highly conserved from yeast to human (Fig. 1a). In addition to this core domain, human MIA40 harbors N- and C-terminal extensions that are not essential for its enzymatic function as demonstrated by complementation experiments in yeast [21, 24]. The N-terminal region of human MIA40 is critical for interaction with apoptosis-inducing factor (AIFM1) [25–27]. AIFM1 thereby facilitates the IMS import of MIA40, which does not contain an MTS [26]. Absence of this interaction results in a hampered mitochondrial import of MIA40, decreased cellular levels of MIA40, and consequently, also reduced levels of MIA40 substrates [25, 26]. However, the relevance of the C-terminal region of MIA40 is unknown.

In this study, we demonstrate that this region ensures efficient import of MIA40 into mitochondria. It is highly negatively charged, and this is an evolutionarily conserved feature. We demonstrated that truncating the C-terminal region did not affect the stability or activity of purified MIA40 in vitro. It did also not affect functionality and localization of MIA40 in intact cells or the stability of the mature folded and oxidized protein in the IMS. Instead, removing the C-terminal region increased the rate of mitochondrial import, however, at the cost of a strongly lowered cytosolic stability of the MIA40 precursor. This lead to mitochondrial import of decreased amounts of MIA40 and lowered cellular steady-state levels of MIA40. Lack of the C-terminal region could be overcome by rerouting MIA40 import and equipping the protein with an MTS. We could transfer the stabilizing properties of the C-terminal region of MIA40 by fusing it to different variants of the IMS-protein COX19. Collectively, this demonstrates that the C-terminal region acts in two ways on the MIA40 precursor: by protecting the MIA40 precursor from proteasomal degradation at the costs of slowing its mitochondrial import and leading to extended posttranslational dwelling times of MIA40 in the cytosol.

## Results

### MIA40 contains a C-terminal region with a conserved highly negative charge

The structure of the core domain of human MIA40 has been solved and exhibits a helix-loop-helix conformation connected by two disulfide bonds formed by the four cysteines of the twin-CX_9_C motif. N-terminal to this core domain that is important for hydrophobic interactions with MIA40 substrates is a small helix that contains the redox-active CPC (Cys-Pro-Cys) motif. In the structure, both the N- and C-terminal regions have not been assessed (protein for structure determination reaches from aa 45-aa 106) [21]. They are predicted to be mostly unstructured (Fig. 1a) and were not considered important for MIA40 function as deletion of the *MIA40* gene in yeast could be complemented by truncated versions of human MIA40 [21, 24]. However, recent work in mammalian cells demonstrated that the most N-terminal 20–30 amino acids are critical for mitochondrial import of MIA40 by AIFM1 highlighting non-conserved differences between human and yeast cells [26].

The function of the C-terminal region of MIA40 consisting of about 45 amino acids after the last cysteine of the structural twin-CX_9_C motif has not been assessed. An in silico analysis of this region indicated an accumulation of negatively charged amino acid residues (16 negatively charged amino acids out of the last 35 amino acids). This anionic character of the C-terminus is present to varying degrees among MIA40 from different species (Additional file 1: Figure S1A). Interestingly, no other twin-CX_n_C protein of the human IMS contains such an extended negative feature at its C-terminus. In human MIA40, these negatively charged amino acids clustered into three sections (Fig. 1a) and we thus decided to assess their importance by generating three truncation variants according to this clustering, either lacking amino acid residues 108–142 (MIA40^Δ108^), 122–142 (MIA40^Δ122^), or 131–142 (MIA40^Δ131^).

### The negative charge of the C-terminal region of MIA40 is critical for stability in intact cells

We first characterized the three MIA40 truncation variants and MIA40 wildtype (MIA40^WT^) in vitro*.* The purified proteins were stable in solution (Additional file 1: Figure S1B). They exhibited very similar spectra in circular dichroism (CD) spectroscopy with an α-helical content of more than 90% which was more than expected from in silico predictions (Additional file 1: Figure S1C). When we analyzed the thermal stability, we confirmed the previously reported high stability of the core domain of MIA40^WT^ for the full-length protein [21] and found the MIA40 truncation variants to be equally stable (Additional file 1: Figure S1D). Likewise, when we tested the stability of the twin-CX_9_C motif of MIA40 towards reducing agents, we found similar stability of MIA40^WT^ and MIA40 truncation variants (Additional file 1: Figure S1E). Next, we assessed the importance of the C-terminal region for MIA40 function. We employed an in vitro reconstituted system [15, 28] and assessed oxidation of the MIA40 substrates, reduced human NDUFA8 and COX19, by oxidized MIA40 variants. All MIA40 variants exhibited the same capacity in oxidizing these substrates (Additional file 1: Figure S1F). Collectively, our data indicate that the negatively charged C-terminal region of human MIA40 is not critical for in vitro stability and activity of the enzyme.

We next analyzed MIA40 variants in cellulo. MIA40^WT^ and truncation variants were all localized to mitochondria (Fig. 1b, Additional file 1: Figure S1G). MIA40^WT^ and MIA40^Δ108^ retained the same semi-oxidized redox state of their CPC active site (Additional file 1: Figure S1H). They were also fully functional as they complemented a MIA40 depletion cell line (Fig. 1c, d) [15]. In the MIA40 depletion cell line, levels of most substrates of the mitochondrial disulfide relay were strongly reduced. Loss of substrates could be complemented by expressing MIA40^WT^ and the truncation variants for 7 days (thereby allowing import, maturation, and accumulation of substrates) but not by expressing the dominant-negative MIA40^C53S^ variant (Fig. 1c). This was also reflected in the improved proliferation of the complemented MIA40 depletion cell line expressing MIA40^WT^ and MIA40^Δ108^ that was absent when expressing MIA40^C53S^ (Fig. 1d). Interestingly, truncation of MIA40 led to a strong reduction in the steady-state levels of MIA40 variants, independent of the presence and position of a tag (Fig. 1e, Additional file 1: Figure S1I,J). Still, all truncated variants complemented the loss of MIA40 in line with previous data based on siRNA experiments that indicated that 10–20% functional MIA40 in the IMS are sufficient to maintain normal levels of its substrates [14]. To test whether the negative charge of the C-terminal region of MIA40 was critical for its stability in cells, we generated additional “charge-change” variants based on MIA40^Δ108^ and MIA40^Δ131^ (Fig. 1f). We thereby found that only the addition of the endogenous negatively charged amino acids increased steady-state levels (Fig. 1g, h). The addition of neutral amino acids only increased steady-state levels to a minor extent compared to the truncated variant, while the addition of positive amino acids even decreased levels (Fig. 1g, h). Thus, C-terminal truncation of MIA40 led to strongly lowered protein levels in cells but did not affect MIA40 localization or activity.

### Truncated MIA40 variants are stabilized by proteasomal inhibition

**Fig. 2.**
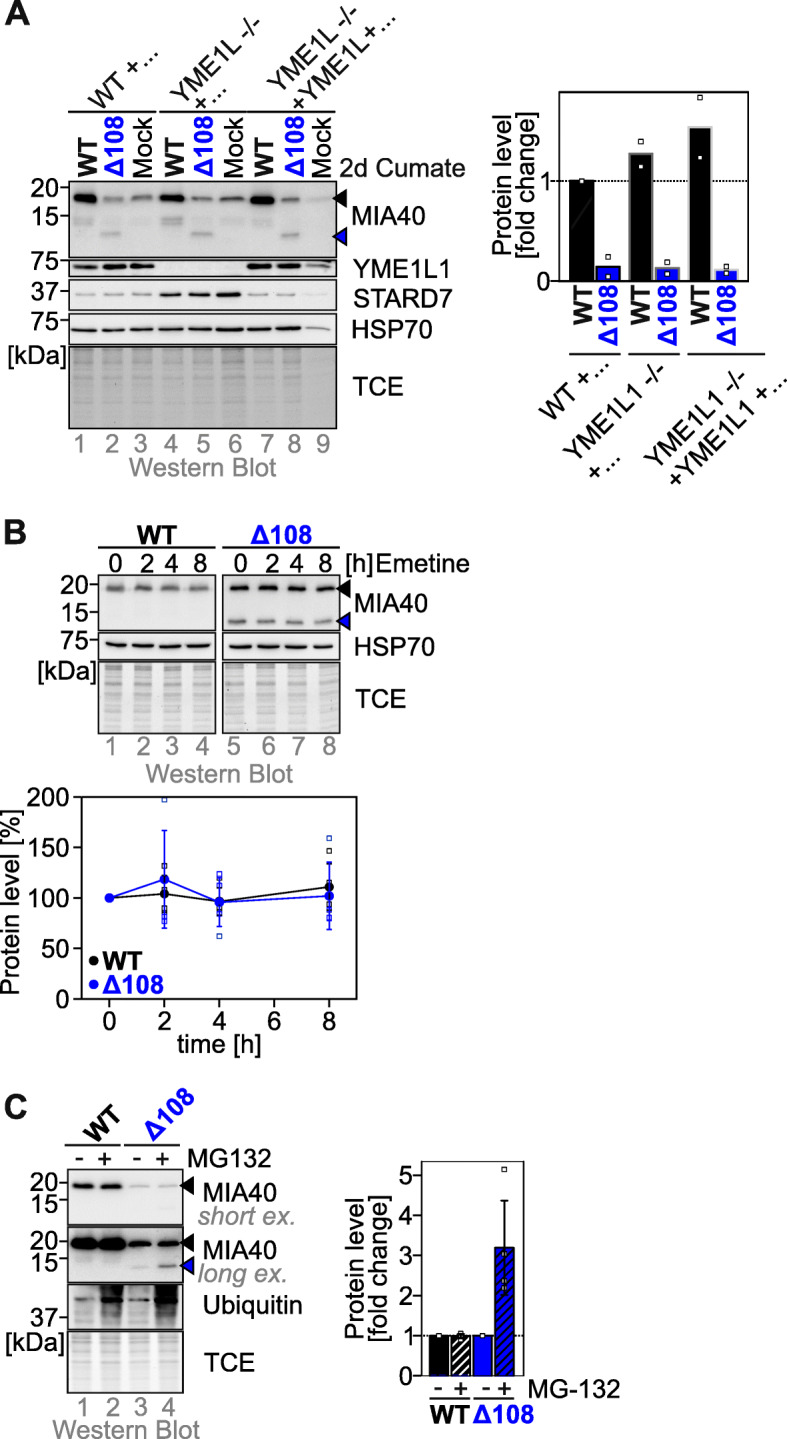
Lowered levels of C-terminally truncated MIA40 can be partially rescued by proteasomal inhibition. **a** Steady-state levels of MIA40 truncation variants in HEK293-based YME1L1 deletion cells. MIA40^Δ108^ and MIA40^WT^ were expressed for 2 days stably, inducibly in YME1L1 knockout in medium containing 30 μg ml^−1^ cumate. Cells were lysed and analyzed by immunoblotting. MIA40^Δ108^ is present at decreased levels compared to MIA40^WT^ and is not stabilized by loss of YME1L1. Quantification using Image Lab. Data from 2 experiments were combined. Black arrowhead, endogenous MIA40; blue arrowhead, signal of MIA40^Δ108^. **b** Emetine chase analyses of truncated MIA40 variants. Protein expression of cells stably expressing MIA40^Δ108^ and MIA40^WT^ was induced for 24 h prior to the experiment with 1 μg ml^−1^ doxycycline. Emetine was added for indicated times, the cells were lysed after 8 h and analyzed by immunoblotting. Mature MIA40^Δ108^ is equally stable as MIA40^WT^. Quantification using Image lab. Data from 3 experiments were combined and standard deviations are presented. Black arrowhead, endogenous MIA40; blue arrowhead, signal of MIA40^Δ108^. **c** Steady-state levels of MIA40^Δ108^ and MIA40^WT^ upon proteasomal inhibition. Expression of MIA40 variants in HEK293 cells was induced using 1 μg ml^−1^ doxycycline for 16 h. Concomitantly, cells were incubated with 1 μM of the proteasome inhibitor MG132. Then, cells were lysed and analyzed by immunoblotting. MIA40^Δ108^ is present at strongly decreased levels compared to MIA40^WT^ but can be partially stabilized by proteasomal inhibition. Quantification using Image lab. Data from 4 experiments were combined and standard deviations are presented. Black arrowhead, endogenous MIA40; blue arrowhead, signal of MIA40^Δ108^

The low levels of truncated MIA40 might stem from accelerated degradation of the protein. To test this, we expressed MIA40^WT^ and MIA40^Δ108^ (the variant on which we focus from now on) in cells lacking the major protease of the IMS, YME1L1, which has previously been shown to degrade disulfide relay substrates [29, 30]. However, when we expressed MIA40^WT^ and MIA40^Δ108^ in YME1L1 knockout cells, we found no changes in their steady-state levels in contrast to the known YME1L1 substrate STARD7 (Fig. 2a, [31]). We next assessed the stability of mature MIA40 variants by emetine chase experiment. Both MIA40^WT^ and MIA40^Δ108^ were stable over a period of 8 h indicating that once MIA40 truncation variants are matured and present in the IMS, they are stable (Fig. 2b, Additional file 2: Figure S2A). This is also in line with the equal stability of the purified MIA40 variants (Additional file 1: Figure S1D). Import of certain disulfide relay substrates competes with their proteasomal degradation [15, 17, 19]. We thus expressed MIA40 variants in the presence of the proteasome inhibitor MG132 and tested their levels. While MIA40^WT^ levels were not affected by MG132, levels of MIA40^Δ108^ increased, however, by far not to wildtype levels (Fig. 2c, Additional file 2: Figure S2B). Since synthesis rates of MIA40^WT^ and MIA40^Δ108^ were very similar (Additional file 2: Figure S2C,D), we reasoned that the C-terminal region of MIA40 might be important during MIA40 import by protecting the precursor during posttranslational import from proteasomal degradation. Notably, treatment of YME1L1 knockout cells with MG132 did not lead to an additional stabilization MIA40 variants supporting the notion that YME1L is not involved in MIA40 degradation (Additional file 2: Figure S2E).

### The C-terminal region protects the cytosolic precursor from proteasomal degradation and slows down mitochondrial import

**Fig. 3.**
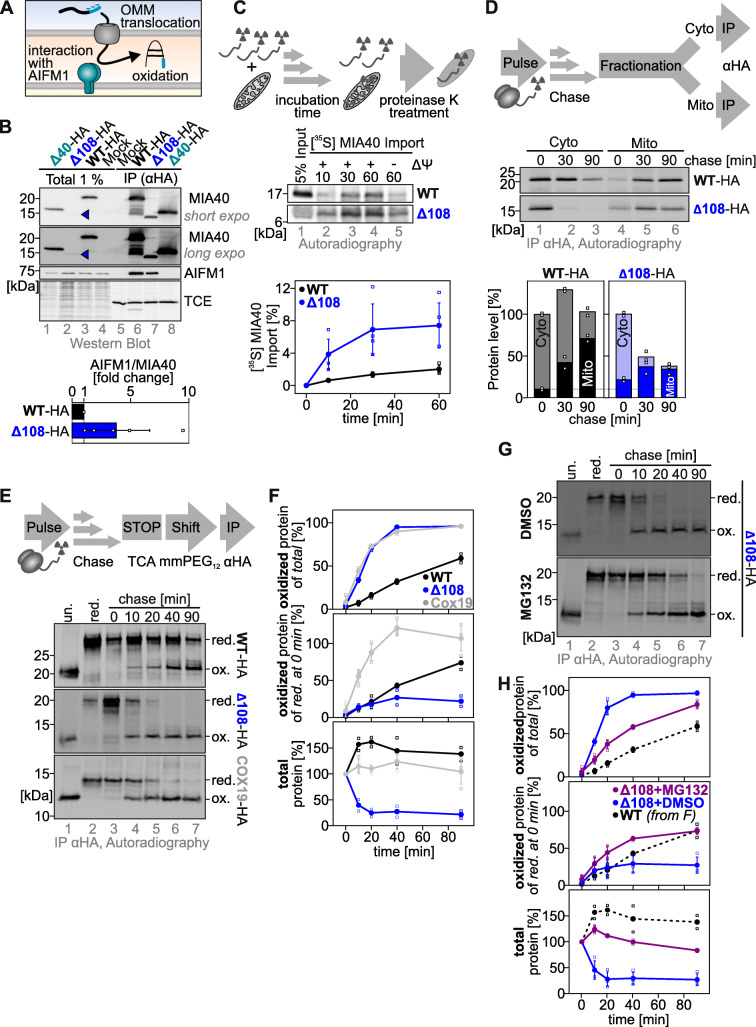
The C-terminal region of MIA40 slows mitochondrial import and concomitantly protects MIA40 from proteasomal degradation. **a** Steps in the biogenesis of MIA40 involve synthesis of the precursor by cytosolic ribosomes, translocation over the OMM, interaction with AIFM1 for translocation, and oxidation by the disulfide relay. **b** Interaction between MIA40 variants and AIFM1. HEK293 cells stably expressing different variants of MIA40-HA (24 h doxycycline) were subjected to native immunoprecipitation (IP) against the HA-tag. Precipitates were analyzed by SDS-PAGE and immunoblotting against the indicated proteins. AIFM1 co-precipitates with both, MIA40^WT^ and MIA40^Δ108^, but not with MIA40^Δ40^. Blue arrowhead, signal of MIA40^Δ108^. Quantification using Image lab. Data from 5 experiments were combined and standard deviations are presented. **c** In organello import assay of MIA40 variants. In vitro translated radioactive proteins were incubated with mitochondria isolated from HEK293 cells. Non-imported proteins were removed by treatment with Proteinase K. Imported proteins were analyzed by reducing SDS-PAGE and autoradiography. Signals were quantified and the amount of imported protein was plotted. MIA40^Δ108^ is imported more rapidly than MIA40^WT^. Quantification using ImageQuantTL. Data from 4 experiments were combined and standard deviations are presented. **d** In cellulo translocation assay. Synthesis of MIA40 variants was induced 1.5 h before the experiment using 1 μg ml^−1^ doxycycline. Cells were pulse-labeled for 5 min with ^35^S-methionine and chased with cold methionine for different times. After the chase, cells were fractionated into a cytosolic and mitochondrial fraction. Both MIA40^Δ108^ and MIA40^WT^ are imported into mitochondria, but less of MIA40^Δ108^. Cytosolic MIA40^WT^ is stable in contrast to cytosolic MIA40^Δ108^. Quantification using ImageQuantTL. Data from 2 experiments were combined and standard deviations are presented. **e**, **f** In cellulo oxidation kinetics assay to follow oxidative folding of MIA40 variants and COX19 in intact cells. Synthesis of MIA40 variants was induced 1.5 h before the experiment using 1 μg/ml doxycycline. Cells were pulse-labeled for 5 min with ^35^S-methionine and chased with cold methionine for different times. The chase was stopped by trichloroacetic acid (TCA) precipitation, and then the lysate was treated with mmPEG_12_ to determine protein redox states, followed by IP against the HA tag. Eluates were analyzed by Tris-Tricine-PAGE and autoradiography. Reduced proteins were modified with mmPEG_12_, whereas oxidized proteins remained unmodified. MIA40 becomes oxidized very slow compared to COX19 with half oxidation times in the range of 90 min. Yet, the reduced cytosolic form of MIA40^WT^ is stable over the course of the experiment. Conversely, reduced MIA40^Δ108^ disappears rapidly. Quantification using ImageQuantTL. Data from 2 to 3 experiments were combined and standard deviations are presented if *n* > 2. Red., reduced; ox., oxidized; un., unmodified. **g**, **h** In cellulo oxidation kinetics assay to follow oxidative folding of MIA40^Δ108^ in the presence or absence of proteasomal inhibition. As **e**, except that oxidation kinetics were performed in the presence or absence of MG132. Reduced MIA40^Δ108^ is strongly stabilized in an import-competent form by MG132. Quantification using ImageQuantTL. Data from 3 experiments were combined (MIA40^WT^, Additional file 3: Figure S3B) and standard deviations are presented. Red., reduced; ox., oxidized; un., unmodified

MIA40 import depends on AIFM1 as an intramitochondrial import receptor that allows for efficient OMM translocation, and oxidative folding of its twin-CX_9_C core (Fig. 3a). The MIA40-AIFM1 interaction takes place via the N-terminal region of MIA40 [26]. We tested whether removal of the C-terminus affected MIA40-AIFM1 interaction. We precipitated a MIA40 variant lacking the first 40 amino acids (MIA40^Δ40^), MIA40^WT^, and MIA40^Δ108^ from cells and then analyzed the precipitates by immunoblot against AIFM1. While MIA40^Δ40^ did not coprecipitate AIFM1, the interaction of C-terminally truncated MIA40 with AIFM1 was not disturbed but increased (Fig. 3b).

We then directly assessed OMM translocation in an in organello import experiment. Interestingly, MIA40^Δ108^ was imported faster than MIA40^WT^ (Fig. 3c, Additional file 3: Figure S3A). Complementing in cellulo radioactive pulse-chase translocation kinetics revealed that MIA40^Δ108^ rapidly disappeared from the cytosolic fraction, but at the 0-min chase time point (i.e., after 5 min pulse), levels of mitochondrial MIA40^Δ108^ were higher than mitochondrial MIA40^WT^ levels (Fig. 3d).

Next, we employed in cellulo oxidation kinetics approaches to assess the oxidation of the two structural disulfide bonds of MIA40 during import. Compared to other disulfide relay substrates, which become oxidized with half-lives clearly shorter than 20 min, MIA40^WT^ was oxidized rather slow with a half time of oxidation close to 90 min (Fig. 3e, f [14];). By comparison, for MIA40^Δ108^, the share of oxidized protein on the total protein at the respective time point increased much quicker than for MIA40^WT^ with kinetics similar to other disulfide relay substrates (Fig. 3e, f [14];). However, this was only in part determined by a faster occurrence of the oxidized form of MIA40^Δ108^ (compare oxidized band at 10 min time points in Fig. 3e, f). The major effect was the rapid drop in levels of reduced (cytosolic) MIA40^Δ108^, which essentially terminated import of MIA40^Δ108^. Thus, the C-terminal tail affects the stability of the reduced (cytosolic) form of MIA40 and influences the speed of OMM translocation.

Next, we tested the influence of proteasomal inhibition by MG132 on oxidative folding kinetics of MIA40^Δ108^. Under these conditions, the reduced precursor was strongly stabilized rendering the import kinetics more similar to the kinetics of MIA40^WT^ (Fig. 3g, h). Conversely, MIA40^WT^ was not affected by MG132 treatment (Additional file 3: Figure S3B). Similarly, depletion of the membrane potential did not disturb MIA40^WT^ oxidation kinetics in contrast to the control protein SOD2 that was affected in its maturation (Additional file 3: Figure S3B,C). In this experiment, we could also confirm that MIA40^Δ108^ oxidation kinetics were indeed more rapid compared to the wildtype (Fig. 3g, h). In summary, MIA40^WT^ is imported into mitochondria exceptionally slowly. MIA40^Δ108^ is imported faster into mitochondria which might explain its increased interaction with its import receptor AIFM1. However, it is rapidly degraded by the proteasome, and this strong destabilization results in accumulation of lower levels of oxidized IMS-localized MIA40.

### Bypassing AIFM1- and disulfide-relay-dependent import increases stability of MIA40^Δ108^

**Fig. 4.**
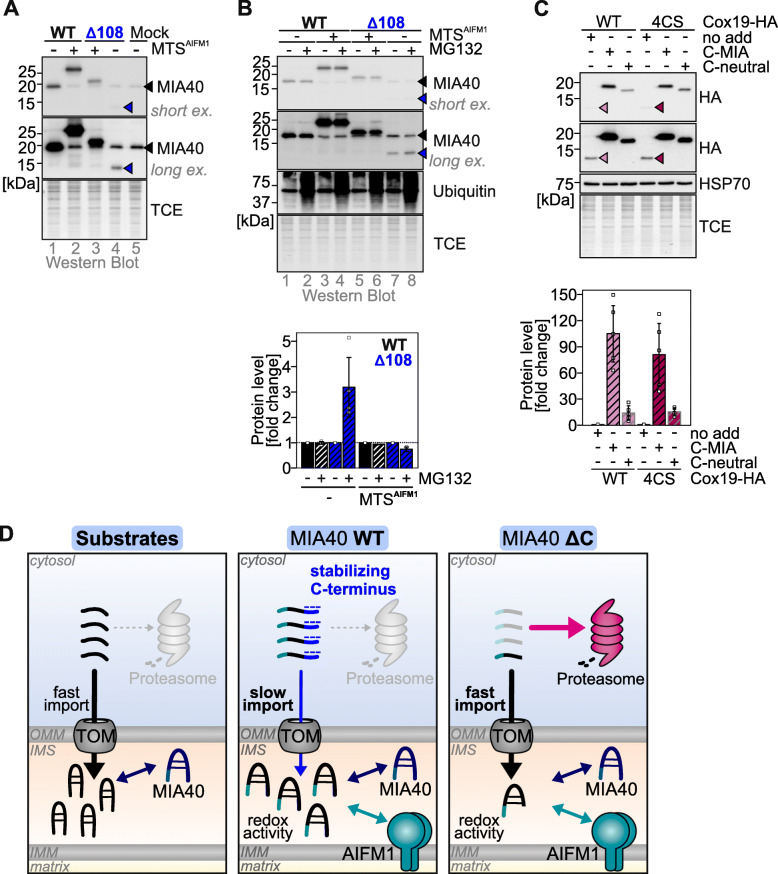
Bypassing the AIFM1- and disulfide relay-dependent import pathway stabilizes MIA40^Δ108^. **a** Steady-state levels of MIA40^Δ108^ and MIA40^WT^ upon rerouting the MIA40 import pathway. Expression of MIA40 variants with and without the N-terminal bipartite mitochondrial targeting sequence of AIFM1 (MTS^AIFM1^) was induced in HEK293 cells using 1 μg ml^−1^ doxycycline for 24 h. MIA40^Δ108^ is stabilized by MTS^AIFM1^. *N* = 2 biological replicates. Black arrowhead, endogenous MIA40; blue arrowhead, signal of MIA40^Δ108^. **b** Like **a**, except that concomitantly to expression (16 h doxycyclin), cells were incubated with 1 μM of the proteasome inhibitor MG132. Then, cells were lysed and analyzed by immunoblotting. MIA40^Δ108^ cannot be further stabilized by MG132 if it is targeted to mitochondria with the MTS^AIFM1^. Quantification using Image lab. Data from 4 experiments were combined and standard deviations are presented. Black arrowhead, endogenous MIA40; blue arrowhead, signal of MIA40^Δ108^. **c** Steady-state levels of COX19 variants extended by the C-terminal stretch of MIA40. Stable HEK293 cells were generated that either expressed COX19^WT^-HA or a COX19 lacking its four cysteines (COX19^4CS^-HA), or fusion proteins of these variants that were extended by either the last 35 amino acids of MIA40 (C-MIA) or the stretch of amino acids in which charged residues were mutated to neutral residues (C-neutral). Expression of COX19 variants was induced using 1 μg ml^−1^ doxycycline for 24 h. Then, cells were lysed and analyzed by immunoblotting. Elongation of COX19 variants increased steady-state levels. The fusion to the endogenous C-terminal stretch of MIA40 thereby had the strongest stabilizing effect. Quantification using Image lab. Data from 5 experiments were combined and standard deviations are presented. Light red arrowhead, COX19^WT^; dark red arrowhead, COX19^4CS^. **d** Model. Compared to other disulfide relay substrates, MIA40 follows an AIFM1- and disulfide-relay dependent import pathway. As a consequence, import and oxidation of MIA40 is a comparatively slow process. Thus, the MIA40 precursor needs to be stabilized in the cytosol. The negatively charged C-terminus of MIA40 contributes to this cytosolic stabilization but also appears to delay MIA40 import

MIA40^WT^ persists for extended times stably in the cytosol. This is possible due to the stabilizing C-terminal region of the protein. The lowered stability of MIA40^Δ108^ in the cytosol should in principle be overcome by facilitating a rapid import into mitochondria. This can be achieved by bypassing the AIFM1- and disulfide relay-dependent import of MIA40 by equipping it with an MTS should result in an even faster import and should stabilize MIA40^Δ108^. We equipped MIA40^WT^ and MIA40^Δ108^ with the MTS of AIFM1, which has been previously used to target functional MIA40 to the IMS [26]. Both proteins became rapidly oxidized indicating efficient IMS import (Additional file 4: Figure S4). When we analyzed the cellular levels of MTS-equipped MIA40 variants, we found that indeed MTS-MIA40^Δ108^ was present at higher levels (Fig. 4a), and levels could not be much further increased by MG132 treatment as was possible with untargeted MIA40^Δ108^ (Fig. 4b). Interestingly, also MIA40^WT^ levels were slightly increased by equipping it with an MTS (Fig. 4a). Next, we tested whether the stabilizing properties of the C-terminal region could be transferred to a different mitochondrial precursor. We thereby either employed the last 35 amino acids of MIA40 or as a control the C-terminal tail with its charged amino acids changed into neutral amino acids. We fused these tails to variants of the IMS protein COX19 (COX19^WT^ and a variant that lacked all four cysteines of COX19 that are important for COX19 import (COX19^4CS^)), which is also a substrate of the mitochondrial disulfide relay and imported in a slow posttranslational fashion. We observed that all fusion proteins were present at increased levels compared to unfused COX19^WT^ and COX19^4CS^ (Fig. 4c) indicating that elongating COX19 already has a stabilizing effect. Importantly, fusion of the negatively charged tail led to an even stronger stabilization of COX19^WT^ and COX19^4CS^ (Fig. 4c) confirming the stabilizing properties of this amino acid stretch. Collectively, our data indicate that the C-terminal region of MIA40 has a profound stabilizing effect on the cytosolic reduced precursor and allows for its continued presence in the cytosol by preventing its degradation.

## Discussion

In this study, we characterized the role of the negatively charged C-terminal region of human MIA40. We found that it has a profound stabilizing effect on the MIA40 precursor in the cytosol and that it slows down mitochondrial import of MIA40 (Fig. 4d). Import of wildtype MIA40 proceeds very slowly, even by the standards of the slow import of other disulfide relay substrates. Extended dwelling of the MIA40 precursor in the cytosol exposes the precursor to increased proteasomal degradation. The addition of the stabilizing C-terminal tail to MIA40 ensures that sufficient amounts of MIA40 reach the IMS, thereby allowing efficient import of this key component of the mitochondrial disulfide relay.

It remains unclear how the negatively charged C-terminal region of MIA40 achieves stabilization of the precursor. Stabilization appears to derive from both the negative charge as well as the simple presence of an amino acid extension. This was best visible upon fusion of the C-terminal tail of MIA40 to COX19. Even the extension with a uncharged tail stabilized COX19. The presence of negative charges in the C-terminal tail further strongly exaggerated this effect (Fig. 4c). This might also explain differences between the different MIA40 truncation variants. The C-terminal region is predicted to be unstructured (Fig. 1a), and charge clustering and absence of hydrophobic residues are often observed in such cases [32, 33]. In our CD analyses, we determined a high α-helical content of MIA40 that would only be in line with an α-helical structure also of the C-terminal region. This implies that despite the high negative charge, the C-terminal regions acquire some structure likely due to the interaction with other parts of MIA40 [34]. This interaction might also contribute to cytosolic protection of MIA40, e.g., because the negative charges cover lysine residues in MIA40 that might become ubiquitylated. Alternatively, the C-terminal region might simply decrease aggregation propensity of the unfolded precursor or it might act as membrane repellant and prevent excessive association with membranes. It might also bind cations like Ca^2+^ or other protein partners that regulate its cytosolic stability.

Is there a reason for the slow import of MIA40 compared to other disulfide relay substrates? It appears that the negatively charged C-terminal tail that is unique to MIA40 does not only stabilize the MIA40 precursor but is also at least in part responsible for slow import. One might therefore speculate that the slow import serves regulatory purposes and has to be interpreted in the light of its special mode of import through AIFM1. AIFM1 is not only the import receptor for MIA40 but also an NADH:ubiquinone oxidoreductase [35]. Once AIFM1 binds NADH, the protein dimerizes, and only in this form does it bind the N-terminal region of MIA40 and import the protein [26]. Thus, AIFM1 might sense the redox state of the Q-pool as well as IMS levels of NADH to match MIA40 import to metabolic demands. The strong stabilization of MIA40 in the cytosol might serve in ensuring a persisting pool of available MIA40 precursor that might be available for import should AIFM1 become dimerized. Additionally, MIA40 might also fulfill as of now unknown extramitochondrial roles. To perform these, the protein likely needs to become folded. To ensure sufficient time for folding, MIA40 needs to become imported slowly and remain stable as a precursor.

In summary, both “unstructured” regions of MIA40 that are specific for higher eukaryotes fulfill non-conserved import functions for the MIA40 precursor: the N-terminal regions interact with the import receptor AIFM1, and the C-terminal region stabilizes the MIA40 precursor during its long dwelling time in the cytosol. Our study also provides insights into the molecular determinants that allow differential precursor recognition during cytosolic surveillance.

## Conclusion

This study provides a detailed characterization of the function of the negatively charged C-terminal region of the IMS oxidoreductase MIA40 and thereby allows insights into the molecular determinants of selective precursor stabilization. We demonstrate that this region is dispensable for MIA40 function in vitro and in intact cells; it takes however a crucial role in stabilizing the MIA40 precursor during its transit from cytosolic ribosomes to mitochondria. This stabilization becomes necessary because of the slow import of MIA40 by its dedicated import receptor AIFM1 that results in an extended residence time of the MIA40 precursor in the cytosol. This stabilization might thereby also allow a regulation of MIA40 import by metabolic fluctuations. AIFM1 is only active in MIA40 import when it binds NADH. Upon loss of NADH from AIFM1, MIA40 import stops. The presence of a stable cytosolic pool of MIA40 precursor would allow rapid resuming of MIA40 import once NADH becomes available again for AIFM1.

## Material and methods

### Plasmids and cell lines

For plasmids and cell lines used in this study, see Additional file 5: Tables S1-S3. All cell lines were cultured in Dulbecco’s modified Eagle’s medium (DMEM) complete containing 4.5 g l^−1^ glucose, 10% fetal bovine serum (FCS), and 500 μg ml^−1^ penicillin/streptomycin at 37 °C under 5% CO_2_. For the generation of stable, doxycycline (DOX)-inducible cell lines, the HEK293 cell line-based Flp-In™ T-REx™-293 cell line was used with the Flp-In™ T-REx™ system (Invitrogen). Positive clones were selected with DMEM complete containing 100 μg ml^−1^ hygromycin and 10 μg ml^−1^ blasticidin. The expression of stable cell lines was induced for at least 16 h with 1 mg ml^−1^ doxycycline. For the stable complementation of the HEK293T MIA40 CRISPR cell line #2 [15], the different MIA40 constructs were inserted into the cumate-inducible PB-CuO-MCS-IRES-GFP-EF1-CymR-Puro vector (System Biosciences). Positive clones were selected with DMEM complete containing 2 μg ml^−1^ puromycin and expression of the cell line was induced with 30 mg ml^−1^ cumate for 5–7 days.

### Western blot analysis and antibodies

For analyzing steady-state protein levels, cells were washed once with ice-cold PBS, harvested in Laemmli buffer (2% SDS, 60 mM Tris-HCl pH 6.8, 10% glycerol, 0.0025% bromophenol blue) containing 50 mM dithiothreitol (DTT), and lysed by boiling for 5 min at 96 °C and sonification (60% amplitude, 5 cycles). Protein samples were analyzed by SDS-PAGE and immunoblotting. The addition of 2,2,2-trichloroethanol (TCE) into the SDS-PAGE gel allowed for visualization of migrated proteins and as a loading control. For antibodies used in this study, see Additional file 5: Table S1.

### Cell treatments with MG132/emetine

To inhibit the proteasome, cells were treated with MG132 (Sigma-Aldrich) or with DMSO as control. For western blot analysis, cells were treated for 16 h with DMEM complete containing 1 μM MG132 and doxycyclin. In pulse or pulse-chase experiments, cells were treated with 5 μM MG132, which was added to all media except for the chase medium. For the inhibition of the cytosolic ribosome, cells were treated for 0, 2, 4, or 8 h with 100 μg ml^−1^ emetine (Sigma-Aldrich).

### In vitro protein expression and purification

Experiments were performed as described in [36]. For recombinant protein expression, MIA40 variants were cloned into the pGEX-6p-1 (GE Healthcare) expression vector containing N-terminal cleavable GST tag. The proteins were heterologously expressed in *E. coli* Rosetta2™ (DE3) cells and lysed by French pressure in lysis buffer (20 mM Tris-HCl pH 7.4, 200 mM NaCl, 0.2 mM PMSF). After clearing centrifugation, GST-fused MIA40 proteins were purified using GSTrap 4B columns (GE Healthcare). The GST-tag was cleaved off using previously immobilized recombinant PreScission protease. Further purification and removal of the GST was achieved via size exclusion chromatography using a Superdex 16/600 75 pg High Load column (GE Healthcare). The proteins were stored in 20 mM TRIS pH 7.2 and 100 mM NaCl at 4 °C.

### Circular dichroism (CD) spectrometry

Prior to the record of the CD spectra, purified MIA40 variants were dialyzed to 100 mM KPi pH 7.2 buffer. For estimation of the change in the extent of secondary structure upon unfolding, a series of 13 spectra (each an accumulation of 5 individual spectra with a data pitch of 1 nm) was recorded in a range of temperature between 40 and 100 °C at 5 °C increments.

The ellipticity *θ*_*d*_ was normalized to the protein concentration (*c*), the number of residues (*nr*), the cuvette diameter (*l*), and the molecular weight (*M*).

$$ {\theta}_{mr}={\theta}_d\cdot \frac{M}{c\cdot l\cdot {n}_r} $$

The resulting molar ellipticity *θ*_*mr*_ was plotted over the wavelength *λ*. For temperature stability determination, spectra were recorded between 40 and 95 °C. For *T*_*M*_ determination, spectra at 206 and 225 nm were assessed.

### In vitro direct redox state assay

To analyze the direct redox state, recombinantly expressed MIA40 proteins were reduced with 2 mM tris(2-carboxyethyl)phosphine (TCEP) for 15 min at either 4 or 96 °C. Water instead of TCEP was added to the steady-state and unmodified samples. Free thiols were modified with the maleimide compound mmPEG_24_ at a final concentration of 2 mM for 1 h at RT. The unmodified controls were incubated with water. Samples were analyzed by SDS-PAGE and Coomassie staining.

### In vitro oxidation kinetic with purified proteins

This assay was performed as described in [15]. The radiolabeled MIA40 substrates human COX19 and NDUFA8 were synthesized in vitro using the TNT Quick Coupled Transcription/Translation System (Promega) under normoxic conditions. 1 mM DTT was added during the synthesis to keep the proteins in a reduced state. Redox state was verified by alkylation assays. The oxidation reaction was started by adding a dilution of 1:40 of the radioactive lysate to 5 μM recombinant MIA40 protein diluted in reaction buffer (20 mM TRIS pH 7.2, 200 mM NaCl, 1 mM EDTA). After indicated times, the reaction was stopped by adding 10% trichloroacetic acid (TCA). After TCA precipitation, the pellets were resolved in Laemmli buffer containing 5 mM mmPEG_24_ and incubated for 1 h at RT. Samples were analyzed by SDS-PAGE and autoradiography.

### In organello import assay

For the isolation of crude mitochondria, HEK293 cells were washed in PBS and resuspended in homogenization buffer (220 mM mannitol, 70 mM sucrose, 5 mM HEPES/KOH pH 7.4, 1 mM EGTA) prior to homogenization with a rotating Teflon potter (Potter S, Braun). The homogenate was cleared of debris and nuclei by centrifugation at 600*g* for 5 min at 4 °C. The supernatant was centrifuged at 8000*g* for 10 min at 4 °C to obtain mitochondrial fractions. Mitochondria were washed in homogenization buffer and 100 μg was taken per import reaction. Radiolabeled precursor proteins were synthesized for 1 h at 30 °C using the SP6 promoter TNT Quick Coupled Transcription/Translation System (Promega) containing 20 μCi [^35^S]-methionine. Import assays were performed as described previously [31]. Briefly, protein import was initiated by incubating precursor protein with crude mitochondria at 30 °C in the presence or absence of CCCP (1 mM). Import was stopped after 10, 30, or 60 min by placing mitochondria on ice. All samples were treated with proteinase K (20 μg ml^−1^) for 20 min to degrade non-imported precursor protein. Mitochondria were then washed in homogenization buffer containing PMSF (1 mM) and resuspended in Laemmli buffer for analysis by SDS-PAGE and autoradiography.

### Immunoprecipitation

For native immunoprecipitation, cells were once washed and then incubated for 10 min at 4 °C in ice-cold PBS containing 20 mM NEM (N-Ethylmaleimide). After sedimentation (5 min, 500×*g*, 4 °C), cells were resuspended in 3 ml native lysis buffer (100 mM NaPi pH 8.1, 1% Triton X-100) per 10 cm plate and incubated for 1 h on ice for efficient native lysis. From here on, the immunoprecipitation was performed the same for the following experiments: native immunoprecipitation, pulse assay, and oxidative protein folding assay and organelle fractionation assay. Cell debris was sedimented (1 h, 20,000×*g*, 4 °C) and the supernatant was subjected to immunoprecipitation with equilibrated monoclonal anti-HA agarose conjugate beads (HA beads; Sigma-Aldrich) at 4 °C overnight under gentle shaking. The samples were washed five times in wash buffer A containing Triton X-100 (100 mM NaPi pH 8.1, 1% Triton X-100, 250 mM NaCl) and once in wash buffer B without Triton X-100 (100 mM NaPi pH 8.1, 250 mM NaCl). Immunoprecipitated proteins were eluated from dried HA beads by adding Laemmli buffer and subsequent boiling for 7 min at 96 °C. Samples were analyzed by SDS-PAGE or Tris-Tricine-PAGE.

### In cellulo pulse assay

Cells were starved in cysteine- and methionine-free medium (Sigma-Aldrich) for 15 min at 37 °C. Newly synthesized proteins were pulse-labeled for 5, 10, 20, and 40 min at 37 °C in cysteine- and methionine-free medium containing 100 μCi ml^−1^ EasyTag EXPRESS 35S Protein Labeling Mix (PerkinElmer). To inhibit the proteasome, the starvating and pulse medium was supplemented with 5 μM MG132 (Sigma-Aldrich) or with DMSO as control. Pulse labeling was stopped by removing the medium and adding ice-cold PBS. After sedimentation (5 min, 500×*g*, 4 °C), the cells were resuspended in 250 μl denaturing lysis buffer A (30 mM Tris pH 8.1, 100 mM NaCl, 5 mM EDTA, 2% SDS) and incubated for 15 min at 96 °C. 750 µl denaturing lysis buffer B (30 mM Tris pH 8.1, 100 mM NaCl, 5 mM EDTA, 2.5% Triton X-100) was added and the samples were incubated for 1 h on ice. From here on, the immunoprecipitation was performed as described above. Samples were analyzed by SDS-PAGE and autoradiography.

### In cellulo oxidation kinetics assay

The assay to follow the oxidative protein folding of proteins in HEK293 cells was performed as described in [14]. Cells were starved in cysteine- and methionine-free medium (Sigma-Aldrich) for 15 min at 37 °C. Newly synthesized proteins were pulse-labeled for 5 min at 37 °C in cysteine- and methionine-free medium containing 200 μCi ml^−1^ EasyTag EXPRESS 35S Protein Labeling Mix (PerkinElmer). To inhibit the proteasome, the starvating and pulse medium was supplemented with 5 μM MG132 (Sigma-Aldrich) or with DMSO as control. Pulse labeling was stopped by removing the medium and adding chase medium containing 20 mM methionine. The chase was performed for 0, 10, 20, 40, and 90 min at 37 °C and stopped by adding ice-cold 8% trichloroacetic acid (TCA). After TCA precipitation, the samples were resolved in modification buffer (0.2 M Tris pH 7.5, 6 M urea, 10 mM EDTA, 2% SDS) and the proteins were modified with a final concentration of 15 mM mmPEG_12_ for 1 h at RT. Reduced control samples were treated with 2 mM Tris (2-carboxyethyl)phosphine (TCEP) for 5 min at 96 °C previous to modification. Oxidized control samples were resolved in modification buffer without mmPEG_12._ After modification, 250 μl denaturing lysis buffer A was added and the samples were incubated or 15 min at 96 °C. Afterward, 750 μl denaturing lysis buffer B was added and the samples were incubated for 1 h on ice. From here on, the immunoprecipitation was performed as described above. Samples were analyzed by Tris-Tricine-PAGE and autoradiography.

### In cellulo translocation assay

To follow the posttranslational import of newly synthesized proteins into mitochondria of HEK293 cells, this assay was performed as the oxidative protein folding assay with the following modifications and as described previously [14]. After the case, cells were fractionated for 30 min at 4 °C in ice-cold fractionation buffer (20 mM 4-(2-hydroxyethyl)-1-pipera- zineethanesulfonic acid [HEPES] pH 7.4, 250 mM sucrose, 50 mM KCl, 2.5 mM MgCl, 1 mM DTT, 0.003% digitonin). By centrifugation at 10,000×*g*, 4 °C, the samples were fractionated into a cytosolic and mitochondrial fraction. The latter was digested for 20 min at 4 °C with ice-cold trypsin buffer (20 mM HEPES, pH 7.4, 250 mM sucrose, 50 mM KCl, 2.5 mM MgCl, 1 mM DTT, 25 μg ml^−1^ trypsin). TCA with a final concentration of 8% was added to all samples. After TCA precipitation and immunoprecipitation, the samples were analyzed by SDS-PAGE and autoradiography.

### In cellulo inverse redox state assay

The inverse redox state assay was performed as described previously [37]. In short, to block reduced thiols, cells were once washed and then incubated for 10 min at 4 °C in ice-cold PBS containing 15 mM NEM (N-Ethylmaleimide). Oxidized controls were pretreated for 10 min at 37 °C in warm PBS containing 10 mM diamide prior to the NEM blockage reaction. Unmodified, min. and max. controls were washed and incubated for 10 min at 4 °C in ice-cold PBS without NEM. Thiol-exchange reactions were stopped by the addition of 8% ice-cold TCA. After TCA precipitation, the samples were resolved in Laemmli buffer containing 5 mM TCEP by sonification and afterward incubated for 15 min at 45 °C. After TCEP reduction, the samples were modified with mmPEG_24_ (15 μM final concentration) for 1 h at room temperature. Min. shift samples were modified with NEM instead. For unmodified samples, the same amount of water was added. Samples were analyzed by Tris-Tricine-PAGE and immunoblotting.

### Immunofluorescence

Immunofluorescence experiments were performed as described in [36]. In short, HEK293 Flp-In T-REx cells expressing HA-tagged MIA40 variants were cultured on poly-L-lysine-coated coverslips. Cells were stained with Mitotracker red (Thermo Fisher) for 1 h. After cell fixation with 4% paraformaldehyde for 15 min, cells were permeabilized with blocking buffer (10 mM HEPES pH 7.4, 3% BSA, 0.3% Triton X-100) for 1 h. Cells were washed and incubated with primary (anti-HA, 3F10, Roche) and secondary antibodies (anti-rat, AlexaFluor 488) for 16 h at 4 °C and 1 h at RT, respectively. After washing in PBS, nuclei were stained with 1 μg ml^−1^ DAPI for 15 min. Coverslips were mounted onto microscope slides using mounting medium (30% glycerol, 12% polyvinyl alcohol, 60 mM TRIS, 2.5% 1,4-diazabicyclo-2,2,2-octan) and dried overnight in the darkness. Cells and pictures were analyzed by confocal fluorescence microscopy (Leica Microsystems TCS SP8; Inverse, DMi 8 CS; PL Apo 63x/1.40 Oil CS2, LAS X) and Fiji, respectively [38].

### Digitonin fractionation

For analyzing the subcellular localization, doxycyclin-induced (16 h) cells were trypsinized and washed in 200 μl ice-cold fractionation buffer (20 mM HEPES pH 7.4, 250 mM Sucrose, 50 mM KCl, 2.5 mM MgCl, 1 mM DTT). The cells were then resuspended in 800 μl fractionation buffer containing either 0, 0.003, 0.005, 0.01, 0.03, 0.05, 0.1, or 0.3% digitonin (PanReac AppliChem). 25 U Benzonase® Nuclease (Sigma-Aldrich) was added for DNA degradation. The samples were incubated for 30 min on ice while being inverted every 5 min. The supernatant and the pellet fraction was then separated by centrifugation at 9000*g* for 10 min at 4 °C. Proteins of the supernatant were precipitated in ice-cold TCA. The pellet was resuspended in 800 μl fractionation buffer containing 25 μg ml^−1^ trypsin and the samples were incubated for 30 min on ice while being inverted every 5 min. The proteins of the pellet fraction were then precipitated in ice-cold TCA. After TCA precipitation, the samples were resolved in modification buffer (0.2 M Tris pH 7.5, 6 M urea, 10 mM EDTA, 2% SDS). The samples were sonicated until the pellets were entirely dissolved, supplemented with Laemmli buffer for analysis by SDS-PAGE and immunoblotting.

### Viability assay

For analyzing the cell viability, cells were adapted and cultured in galactose-containing medium (DMEM supplemented with 4.5 g l^−1^ galactose, 2 mM L-glutamine, 1 mM sodium pyruvate, 1× nonessential amino acids, 50 mg l^−1^ uridine, 10% FCS and 500 μg ml^−1^ penicillin/streptomycin) at 37 °C under 5% CO2. Twenty-four hours after the cell seeding (day 0), the viability of the cells was determined by treating the cells for 1 h at 37 °C with the PrestoBlue™ Cell Viability Reagent (Invitrogen by Thermo Fisher). The fluorescence was measured at excitation and emission wavelengths of 560 and 590 nm, respectively. After the measurement, the cells were cultured in galactose-containing medium containing 30 mg ml^−1^ cumate. Control cells were treated without cumate. After 1, 3, 5, and 7 days the viability of the cells was measured again. Fluorescence values of cells treated with cumate were divided by fluorescence values of cells treated without cumate.

### Conservation analysis and sequence logo

The primary sequence alignment of the MIA40 C-terminus (isoform 1, CHCHD4.1) was performed by using the plastp protein-protein BLAST algorithm (NCBI) and full-length protein sequences provided by NCBI. The alignment and sequence logo were generated by Jalview [39] with the help of the algorithm of ClustalOWS [40]. Sequences from 86 species were aligned for Fig. 1a. The conservation is depicted as a sequence logo where the relative size of the respective letters indicates the frequency in the sequences. The percentage of negatively charged amino acids was calculated and the presence of patches of at least four negatively charged aa in a row was analyzed for the C-termini. Protein sequences of 36 MIA40 substrates were analyzed manually for the occurrence of negatively charged amino acids.

### Three-dimensional structure and secondary structure prediction

The three-dimensional NMR structure of the core of MIA40 [2K3J] [21] was illustrated by using the protein data bank RCSB PDB. The secondary structure of the N- and C-terminus of MIA40 was predicted with JPred4 [41]. Arrows represent beta-sheets and boxes stand for alpha-helices.

### Quantification and statistical analysis

Immunoblot signals and intensities of autoradiograms were quantified using Image Lab Software (Bio-Rad Laboratories GmbH) and ImageQuant TL (GE Healthcare), respectively. Error bars in figures represent standard deviation and the *n*-number of experiments is noted in each figure legend.

## Supplementary information

**Additional file 1: Figure S1.** The negative charges in the C-terminal region of MIA40 are conserved and C-terminal truncation of MIA40 does not affect its stability and activity in vitro.

**Additional file 2: Figure S2.** In intact cells, C-terminal truncated MIA40 variants can be stabilized by proteasomal inhibition.

**Additional file 3: Figure S3.** In intact cells, MIA40^WT^ is very slowly imported into mitochondria independently of the membrane potential.

**Additional file 4: Figure S4.** In intact cells, MTS^AIFM1^-MIA40 variants are rapidly oxidized.

**Additional file 5: Tables S1-S3. Table S1.** Reagents and Ressources, **Table S2.** Cell lines, and **Table S3.** Oligonucleotides.

## Data Availability

All data generated during this study are included in either the manuscript or its additional files. Material is available upon request to the corresponding author.

## References

[1] Habich M, Salscheider SL, Riemer J (2019). Cysteine residues in mitochondrial intermembrane space proteins: more than just import. Br J Pharmacol.

[2] Herrmann JM, Riemer J (2010). The intermembrane space of mitochondria. Antioxid Redox Signal.

[3] Ahola S, Langer T, MacVicar T. Mitochondrial Proteolysis and Metabolic Control. Cold Spring Harb Perspect Biol. 2019;11(7):1–19. 10.1101/cshperspect.a033936 PMC6601461 30670467

[4] Modjtahedi N, Tokatlidis K, Dessen P, Kroemer G (2016). Mitochondrial proteins containing coiled-coil-helix-coiled-coil-helix (CHCH) domains in health and disease. Trends Biochem Sci.

[5] Wasilewski M, Chojnacka K, Chacinska A (2017). Protein trafficking at the crossroads to mitochondria. Biochim Biophys Acta Mol Cell Res.

[6] Backes S, Herrmann JM (2017). Protein translocation into the Intermembrane space and matrix of mitochondria: mechanisms and driving forces. Front Mol Biosci.

[7] Chacinska A, Guiard B, Muller JM, Schulze-Specking A, Gabriel K, Kutik S, Pfanner N (2008). Mitochondrial biogenesis, switching the sorting pathway of the intermembrane space receptor Mia40. J Biol Chem.

[8] Endo T, Yamano K (2009). Multiple pathways for mitochondrial protein traffic. Biol Chem.

[9] Erdogan AJ, Riemer J (2017). Mitochondrial disulfide relay and its substrates: mechanisms in health and disease. Cell Tissue Res.

[10] Cavallaro G (2010). Genome-wide analysis of eukaryotic twin CX9C proteins. Mol BioSyst.

[11] Longen S, Bien M, Bihlmaier K, Kloeppel C, Kauff F, Hammermeister M, Westermann B, Herrmann JM, Riemer J (2009). Systematic analysis of the twin cx(9)c protein family. J Mol Biol.

[12] Milenkovic D, Ramming T, Muller JM, Wenz LS, Gebert N, Schulze-Specking A, Stojanovski D, Rospert S, Chacinska A (2009). Identification of the signal directing Tim9 and Tim10 into the intermembrane space of mitochondria. Mol Biol Cell.

[13] Sideris DP, Petrakis N, Katrakili N, Mikropoulou D, Gallo A, Ciofi-Baffoni S, Banci L, Bertini I, Tokatlidis K (2009). A novel intermembrane space-targeting signal docks cysteines onto Mia40 during mitochondrial oxidative folding. J Cell Biol.

[14] Fischer M, Horn S, Belkacemi A, Kojer K, Petrungaro C, Habich M, Ali M, Kuttner V, Bien M, Kauff F (2013). Protein import and oxidative folding in the mitochondrial intermembrane space of intact mammalian cells. Mol Biol Cell.

[15] Habich M, Salscheider SL, Murschall LM, Hoehne MN, Fischer M, Schorn F, Petrungaro C, Ali M, Erdogan AJ, Abou-Eid S (2019). Vectorial import via a metastable disulfide-linked complex allows for a quality control step and import by the mitochondrial disulfide relay. Cell Rep.

[16] Habich M, Riemer J. Stop wasting protein-Proteasome inhibition to target diseases linked to mitochondrial import. EMBO Mol Med. 2019;11(5):1–3. 10.15252/emmm.201910441 PMC6505575 30944106

[17] Bragoszewski P, Gornicka A, Sztolsztener ME, Chacinska A (2013). The ubiquitin-proteasome system regulates mitochondrial intermembrane space proteins. Mol Cell Biol.

[18] Mohanraj K, Wasilewski M, Beninca C, Cysewski D, Poznanski J, Sakowska P, Bugajska Z, Deckers M, Dennerlein S, Fernandez-Vizarra E, et al. Inhibition of proteasome rescues a pathogenic variant of respiratory chain assembly factor COA7. EMBO Mol Med. 2019;11(5):1–21. 10.15252/emmm.201809561 PMC6505684 30885959

[19] Kowalski L, Bragoszewski P, Khmelinskii A, Glow E, Knop M, Chacinska A. Determinants of the cytosolic turnover of mitochondrial intermembrane space proteins. BMC Biol. 2018;16(1):66.1–19. 10.1186/s12915-018-0536-1 PMC6013907 29929515

[20] Banci L, Bertini I, Cefaro C, Cenacchi L, Ciofi-Baffoni S, Felli IC, Gallo A, Gonnelli L, Luchinat E, Sideris D (2010). Molecular chaperone function of Mia40 triggers consecutive induced folding steps of the substrate in mitochondrial protein import. Proc Natl Acad Sci U S A.

[21] Banci L, Bertini I, Cefaro C, Ciofi-Baffoni S, Gallo A, Martinelli M, Sideris DP, Katrakili N, Tokatlidis K (2009). MIA40 is an oxidoreductase that catalyzes oxidative protein folding in mitochondria. Nat Struct Mol Biol.

[22] Kawano S, Yamano K, Naoe M, Momose T, Terao K, Nishikawa S, Watanabe N, Endo T (2009). Structural basis of yeast Tim40/Mia40 as an oxidative translocator in the mitochondrial intermembrane space. Proc Natl Acad Sci U S A.

[23] Peleh V, Cordat E, Herrmann JM. Mia40 is a trans-site receptor that drives protein import into the mitochondrial intermembrane space by hydrophobic substrate binding. Elife. 2016;5:1–19. 10.7554/eLife.16177 PMC4951193 27343349

[24] Grumbt B, Stroobant V, Terziyska N, Israel L, Hell K (2007). Functional characterization of Mia40p, the central component of the disulfide relay system of the mitochondrial intermembrane space. J Biol Chem.

[25] Meyer K, Buettner S, Ghezzi D, Zeviani M, Bano D, Nicotera P (2015). Loss of apoptosis-inducing factor critically affects MIA40 function. Cell Death Dis.

[26] Hangen E, Feraud O, Lachkar S, Mou H, Doti N, Fimia GM, Lam NV, Zhu C, Godin I, Muller K (2015). Interaction between AIF and CHCHD4 regulates respiratory chain biogenesis. Mol Cell.

[27] Petrungaro C, Zimmermann KM, Kuttner V, Fischer M, Dengjel J, Bogeski I, Riemer J (2015). The Ca(2+)-dependent release of the Mia40-induced MICU1-MICU2 Dimer from MCU regulates mitochondrial Ca(2+) uptake. Cell Metab.

[28] Bien M, Longen S, Wagener N, Chwalla I, Herrmann JM, Riemer J (2010). Mitochondrial disulfide bond formation is driven by intersubunit electron transfer in Erv1 and proofread by glutathione. Mol Cell.

[29] Baker MJ, Mooga VP, Guiard B, Langer T, Ryan MT, Stojanovski D (2012). Impaired folding of the mitochondrial small TIM chaperones induces clearance by the i-AAA protease. J Mol Biol.

[30] Schreiner B, Westerburg H, Forne I, Imhof A, Neupert W, Mokranjac D (2012). Role of the AAA protease Yme1 in folding of proteins in the intermembrane space of mitochondria. Mol Biol Cell.

[31] MacVicar T, Ohba Y, Nolte H, Mayer FC, Tatsuta T, Sprenger HG, Lindner B, Zhao Y, Li J, Bruns C (2019). Lipid signalling drives proteolytic rewiring of mitochondria by YME1L. Nature.

[32] Mao AH, Crick SL, Vitalis A, Chicoine CL, Pappu RV (2010). Net charge per residue modulates conformational ensembles of intrinsically disordered proteins. Proc Natl Acad Sci U S A.

[33] Tedeschi G, Salladini E, Santambrogio C, Grandori R, Longhi S, Brocca S (2018). Conformational response to charge clustering in synthetic intrinsically disordered proteins. Biochim Biophys Acta Gen Subj.

[34] Uversky VN (2015). Functional roles of transiently and intrinsically disordered regions within proteins. FEBS J.

[35] Elguindy MM, Nakamaru-Ogiso E (2015). Apoptosis-inducing factor (AIF) and its family member protein, AMID, are rotenone-sensitive NADH:ubiquinone oxidoreductases (NDH-2). J Biol Chem.

[36] Friederich MW, Erdogan AJ, Coughlin CR, Elos MT, Jiang H, O'Rourke CP, Lovell MA, Wartchow E, Gowan K, Chatfield KC (2017). Mutations in the accessory subunit NDUFB10 result in isolated complex I deficiency and illustrate the critical role of intermembrane space import for complex I holoenzyme assembly. Hum Mol Genet.

[37] Erdogan AJ, Ali M, Habich M, Salscheider SL, Schu L, Petrungaro C, Thomas LW, Ashcroft M, Leichert LI, Roma LP (2018). The mitochondrial oxidoreductase CHCHD4 is present in a semi-oxidized state in vivo. Redox Biol.

[38] Schindelin J, Arganda-Carreras I, Frise E, Kaynig V, Longair M, Pietzsch M, et al. Fiji: an open-source platform for biological-image analysis. Nat Methods. 2012;9(7):676–82. 10.1038/nmeth.2019. 10.1038/nmeth.2019 PMC3855844 22743772

[39] Waterhouse AM, Procter JB, Martin DM, Clamp M, Barton GJ (2009). Jalview version 2--a multiple sequence alignment editor and analysis workbench. Bioinformatics.

[40] Sievers F, Higgins DG (2018). Clustal omega for making accurate alignments of many protein sequences. Protein Sci.

[41] Drozdetskiy A, Cole C, Procter J, Barton GJ (2015). JPred4: a protein secondary structure prediction server. Nucleic Acids Res.

