# Supplementary material for: The C-terminal region of the oxidoreductase MIA40 stabilizes its cytosolic precursor during mitochondrial import
Source: BMC Biol. 2020 Aug 6;18:96. doi: 10.1186/s12915-020-00824-1 (PMC7412830; doi:10.1186/s12915-020-00824-1)
Supplement: Supplementary file 1 — Additional file 1: Figure S1. The negative charges in the C-terminal region of MIA40 are conserved and C-terminal truncation of MIA40 does not affect its stability and activity in vitro. [file 12915_2020_824_MOESM1_ESM.pdf]

ADDITIONAL FILES 1-4 - FIGURES

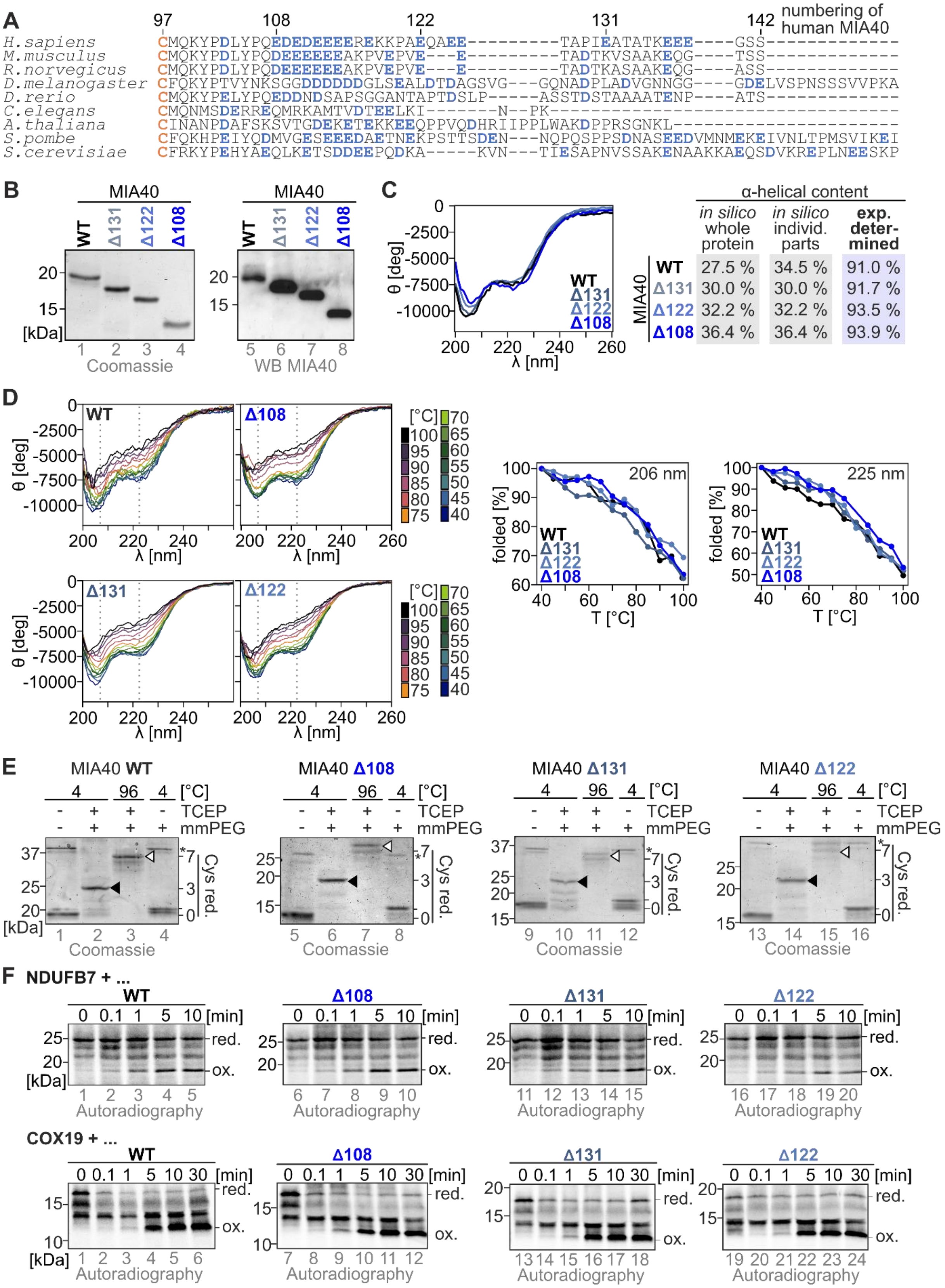

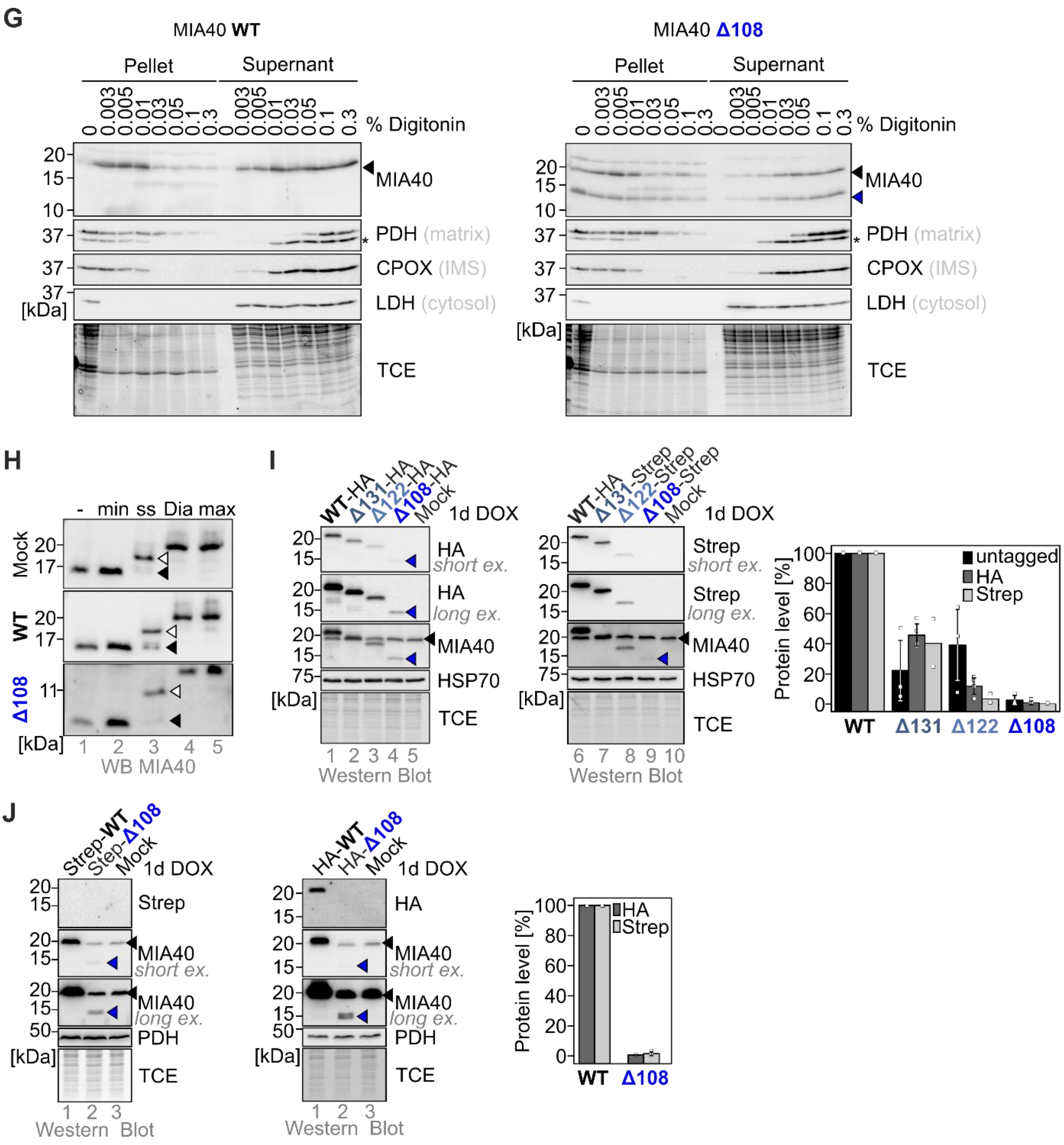

ADDITIONAL FILE 1: Figure S1. The negative charges in the C-terminal region of MIA40 are conserved and C-terminal truncation of MIA40 does not affect its stability and activity *in vitro*.

(A) Alignment of MIA40 from different species. Indicated are negatively charged amino acid residues in the C-terminal region after the last cysteines residues of the core twin-CX_9C motif.

(B) Purification gel and immunoblot analysis of MIA40 truncation variants. Three truncation variants of MIA40 (Δ108, Δ122, Δ131) and the wildtype (WT) were purified to homogeneity from *E. coli*. These data also indicate that the different MIA40 truncation variants are equally reactive with the MIA40 antibody that we use throughout this study.

(C) CD spectra of truncation variants. Calculated and experimentally derived α-helical content of MIA40 truncation variants. We predicted the α-helical content of the MIA40 variants either for the whole protein (*in silico* whole protein) or for each part (N-terminal region, core region, C-terminal region) individually and then combined this to an overall figure for the different MIA40 variants (*in silico* individ. parts). Purified MIA40 variants were analyzed using CD spectroscopy.

(D) Analysis of thermal unfolding of MIA40 truncation variants using CD spectroscopy.

(E) Stability of disulfide bonds in purified MIA40 variants. MIA40 contains seven cysteines of which four are present in structural disulfide bonds and two are present in a labile redox-active disulfide bond. To assess their stability, we employed treatment with the reductant TCEP at different temperatures followed by thiol modification with mmPEG24. The structural disulfides in MIA40 variants are stable against treatment with the reductant TCEP at 4 °C (only three cysteine residues can be modified, black arrow head). Upon treatment with TCEP at 96 °C the structural disulfide bonds open (white arrowhead, 7 cysteines). Asterisk, background band.

(F) *In vitro* oxidation kinetics assay of human COX19 and NDUFB7 using purified MIA40 variant. Recombinant expressed and purified human MIA40 was added to ^{35}S-labeled COX19 or NDUFB7 to allow disulfide exchange reactions for the indicated times. Reactions were stopped by rapid acidification via addition of trichloroacetic acid (TCA). Lysates were treated with mmPEG24 to determine protein redox states, followed by SDS-PAGE and autoradiography. Reduced COX19 and reduced NDUFB7 are modified with four mmPEG24, whereas the oxidized proteins remain unmodified. All MIA40 variants efficiently catalyze oxidation of disulfide bond formation.

(G) Cell fractionation by digitonin titration. HEK293 cells stably expressing MIA40^{WT} or MIA40$^{\Delta108}$ were subjected to fractionation by differential digitonin lysis and centrifugation (expression with 1 µg ml^{-1} doxycyclin for 24 h). Pyruvate dehydrogenase (PDH, coproporphyrinogen oxidase (CPOX) and lactate dehydrogenase (LDH) served as marker proteins for matrix, IMS and cytosol, respectively. MIA40^{WT} or MIA40$^{\Delta108}$ behave similar to the IMS marker. Asterisk indicates the CPOX signal from a previous decoration.

(H) Inverse shift assay to determine the MIA40 redox state. To determine the redox state of MIA40 at steady state (ss), cells were incubated for 24 h with 0.1 or 1 µg ml^{-1} doxycycline prior to the experiment. Intact cells were treated with NEM to stop all thiol-disulfide exchange reactions and trap the redox state of MIA40. Then, cells were lysed and lysates were treated at 4 °C with TCEP. Previously oxidized cysteines in the CPC motif are now accessible to modification with mmPEG24.As controls, unmodified samples (min) and samples without NEM pretreatment (max), as well as samples for which cells were diamide-pretreated (Dia), were loaded. MIA40^{WT} and MIA40$^{\Delta108}$ have a similar semi-oxidized redox state of their CPC motifs. N = 3 biological replicates.

(I,J) Steady state levels of C- (**I**) and N- (**J**) terminally tagged MIA40 truncation variants and MIA40^{WT}. As **Figure 1E**, except that HEK293 cells stably and inducibly expressing different tagged MIA40 variants were analyzed. Tags did not interfere with destabilization by MIA40 truncation. Quantification using Image lab. Data from at least 2 experiments were combined and standard deviations are presented if n>2. DOX, doxycyclin
