# Supplementary material for: The C-terminal region of the oxidoreductase MIA40 stabilizes its cytosolic precursor during mitochondrial import
Source: BMC Biol. 2020 Aug 6;18:96. doi: 10.1186/s12915-020-00824-1 (PMC7412830; doi:10.1186/s12915-020-00824-1)
Supplement: Supplementary file 2 — Additional file 2: Figure S2. In intact cells, C-terminal truncated MIA40 variants can be stabilized by proteasomal inhibition. [file 12915_2020_824_MOESM2_ESM.pdf]

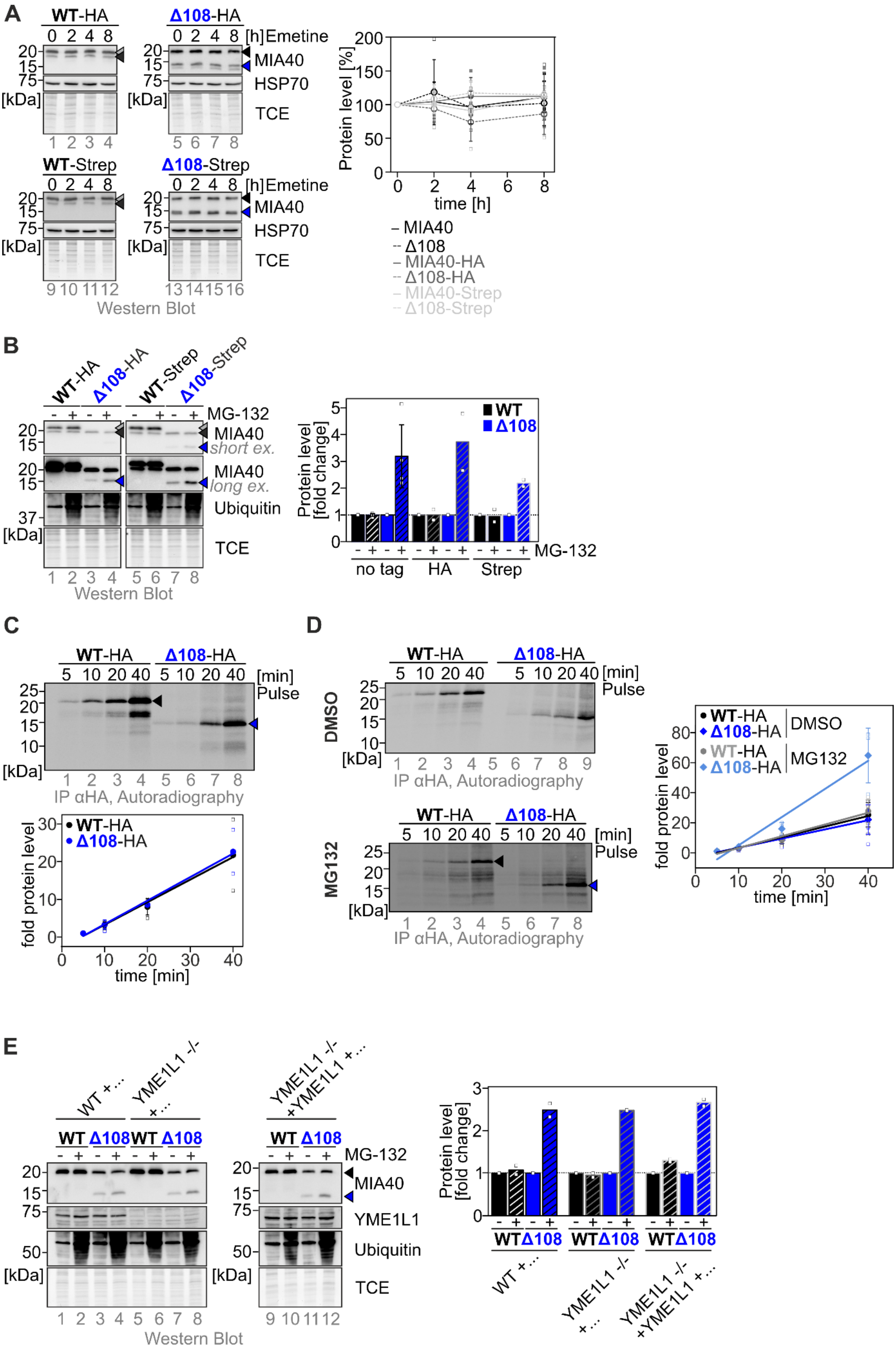
A
WT-HA
Δ108-HA
0 2 4 8 [h]Emetine
MIA40
HSP70
TCE
[kDa]
1 2 3 4
5 6 7 8
WT-Strep
Δ108-Strep
9 10 11 12
13 14 15 16
Western Blot
Protein level [%]
time [h]
– MIA40
-- Δ108
– MIA40-HA
-- Δ108-HA
– MIA40-Strep
-- Δ108-Strep
B
WT-HA
Δ108-HA
WT-Strep
Δ108-Strep
MG-132
MIA40
short ex.
long ex.
Ubiquitin
TCE
1 2 3 4 5 6 7 8
Western Blot
Protein level [fold change]
WT
Δ108
no tag
HA
Strep
C
WT-HA
Δ108-HA
5 10 20 40 [min] Pulse
IP αHA, Autoradiography
fold protein level
time [min]
D
DMSO
MG132
IP αHA, Autoradiography
WT-HA
Δ108-HA
fold protein level
time [min]
E
WT + ...
YME1L1 -/- + ...
YME1L1 -/- +YME1L1 + ...
WT Δ108
MG-132
MIA40
YME1L1
Ubiquitin
TCE
1 2 3 4 5 6 7 8
9 10 11 12
Western Blot
Protein level [fold change]

ADDITIONAL FILE 2: Figure S2. In intact cells, C-terminal truncated MIA40 variants can be stabilized by proteasomal inhibition.

(A) Emetine chase analyses of truncated MIA40 variants. As **Figure 2B** except that tagged MIA40 variants were expressed (1 µg ml^{-1} doxycyclin for 24 h). Mature $MIA40^{\Delta108}$ is equally stable as $MIA40^{WT}$ independently of the presence of a tag. Quantification using Image Lab. Data from at least 2 experiments (HA tagged: n= 3, Strep tagged: n=2) were combined and standard deviations are presented if n>2. Black arrowhead, endogenous MIA40; gray arrowhead, MIA40-HA; blue arrowhead, signal of $MIA40^{\Delta108}$

(B) Steady state levels of $MIA40^{\Delta108}$ and $MIA40^{WT}$ upon proteasomal inhibition. As **Figure 2C** except that tagged MIA40 variants were expressed (1 µg ml^{-1} doxycyclin and 1 µM MG132 for 16 h). $MIA40^{\Delta108}$ is present at strongly decreased levels compared to $MIA40^{WT}$ but can be partially stabilized by proteasomal inhibition independently of the presence of a tag. Quantification using Image Lab. Data from (HA tagged: n= 2, Strep tagged: n=2) experiments were combined and standard deviations are presented if n>2. Black arrowhead, endogenous MIA40; gray arrowhead, MIA40-HA; blue arrowhead, signal of $MIA40^{\Delta108}$

(C) Pulse analysis of MIA40 variant synthesis. HEK293 cells stably and inducibly expressing $MIA40^{WT}$-HA and $MIA40^{\Delta108}$-HA were pulse-labeled with ^{35}S-methionine for different times. Cells were lysed and MIA40 variants isolated by immunoprecipitation against the HA tag. Eluates were analyzed by SDS-PAGE and autoradiography. Synthesis of both variants followed similar kinetics although absolute levels were higher for $MIA40^{WT}$ indicating degradation of $MIA40^{\Delta108}$ during the radioactive pulse.. Quantification using ImageQuantTL. Data from 2-3 experiments were combinedand standard deviations are presented if n>2. Black arrowhead, wildtype MIA40; blue arrowhead, signal of $MIA40^{\Delta108}$

(D) Pulse analysis of MIA40 variant synthesis upon proteasomal inhibition. Experiment was performed as in **(C)**, except that cells were treated with MG132 or DMSO. The wildtype is not stabilized by MG132 treatment. $MIA40^{\Delta108}$ became stabilized upon MG132 treatment indicating that already during the radioactive pulse degradation takes place. Quantification using Image Lab. Data from 2-3 experiments were combined and standard deviations are presented if n>2. Black arrowhead, wildtype MIA40; blue arrowhead, signal of $MIA40^{\Delta108}$

(E) Steady state levels of MIA40 truncation variants in HEK293-based YME1L deletion cells upon proteasomal inhibition. The experiment was performed as in **Figure 2A** except that cells were treated with MG132 or DMSO (1 µg ml^{-1} doxycyclin and 1 µM MG132 for 16 h). $MIA40^{\Delta108}$ is present at decreased levels compared to $MIA40^{WT}$. It is stabilized by MG132 treatment but not by loss of YME1L. Combination of both MG132 and loss of YME1L did not further increase MIA40 levels. Quantification using Image Lab. Data from 2 experiments were combined. Black arrowhead, wildtype MIA40; blue arrowhead, signal of $MIA40^{\Delta108}$
