# Supplementary material for: The C-terminal region of the oxidoreductase MIA40 stabilizes its cytosolic precursor during mitochondrial import
Source: BMC Biol. 2020 Aug 6;18:96. doi: 10.1186/s12915-020-00824-1 (PMC7412830; doi:10.1186/s12915-020-00824-1)
Supplement: Supplementary file 3 — Additional file 3: Figure S3. In intact cells, MIA40WT is very slowly imported into mitochondria independently of the membrane potential. [file 12915_2020_824_MOESM3_ESM.pdf]

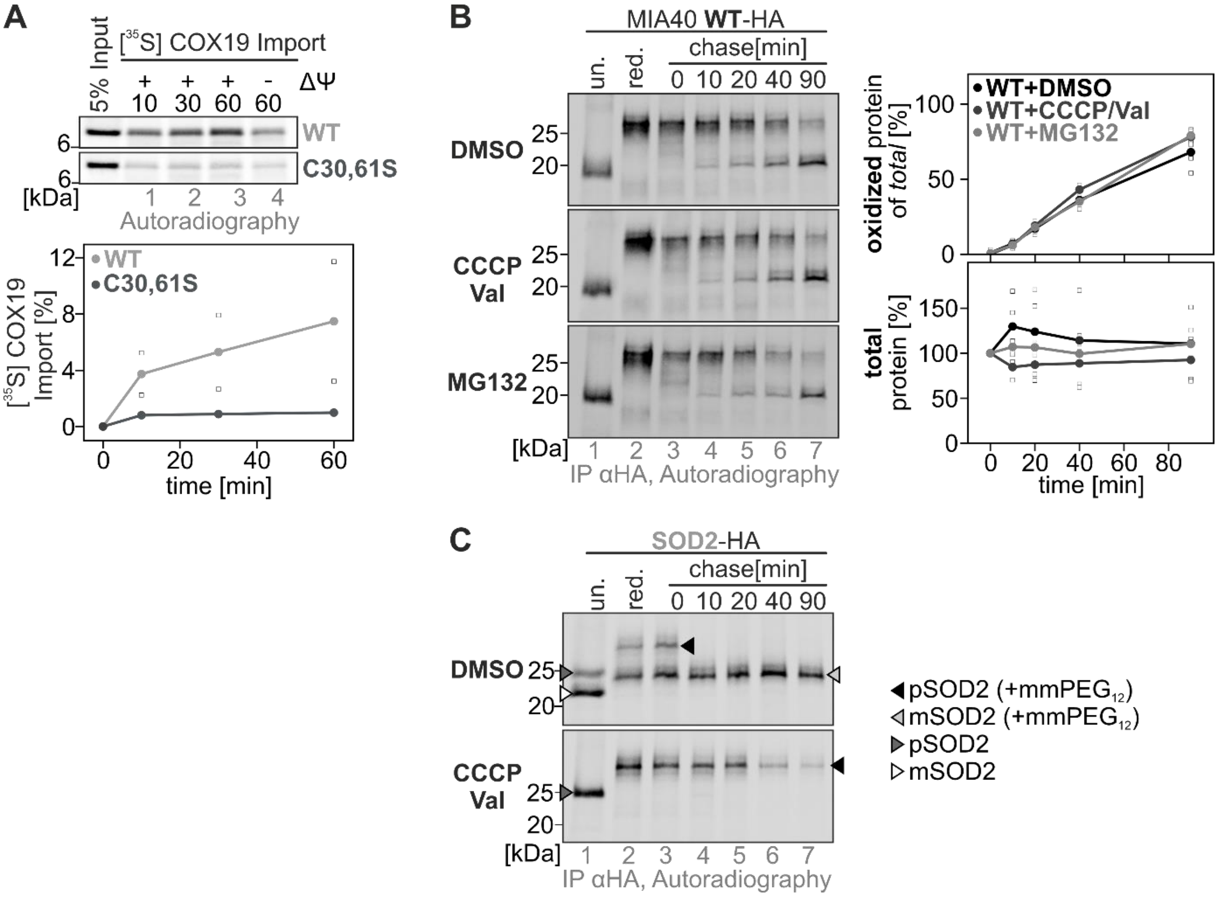

ADDITIONAL FILE 3: Figure S3. In intact cells, MIA40^{WT} is very slowly imported into mitochondria independently of the membrane potential.

(A) Control *in organello* import assay to follow import of the MIA40 substrate and IMS protein COX19 and a variant lacking all cysteines that cannot be imported into mitochondria. Experiment was performed as described in **Figure 3C**. As reported previously, COX19 import is sensitive to loss of its cysteines and lack of the membrane potential. Quantification using ImageQuantTL. Data from 2 experiments were combined.

(B) Control *in cellulo* oxidation assay to follow oxidative folding of MIA40^{WT}. Performed as described in **Figure 3E**, except that the influence of MG132 (proteasome inhibition) and CCCP/valinomycin (membrane potential depletion) was assessed. MIA40^{WT} import is not influenced by CCCP/Valinomycin or MG132. Quantification using ImageQuantTL. Data from 2 experiments were combined.

(C) Control *in cellulo* oxidation assay to follow processing of SOD2. Experiment was performed as in **Figure 3E**. While the SOD2 precursor is rapidly processed under unperturbed conditions, depletion of the membrane potential prevents processing. This experiment serves as control to confirm the depletion of the membrane potential in the import assays as performed in **(A)**, **(B)** and **Figure 3C**.
