# Supplementary material for: The C-terminal region of the oxidoreductase MIA40 stabilizes its cytosolic precursor during mitochondrial import
Source: BMC Biol. 2020 Aug 6;18:96. doi: 10.1186/s12915-020-00824-1 (PMC7412830; doi:10.1186/s12915-020-00824-1)
Supplement: Supplementary file 4 — Additional file 4: Figure S4. In intact cells, MTSAIFM1-MIA40 variants are rapidly oxidized. [file 12915_2020_824_MOESM4_ESM.pdf]

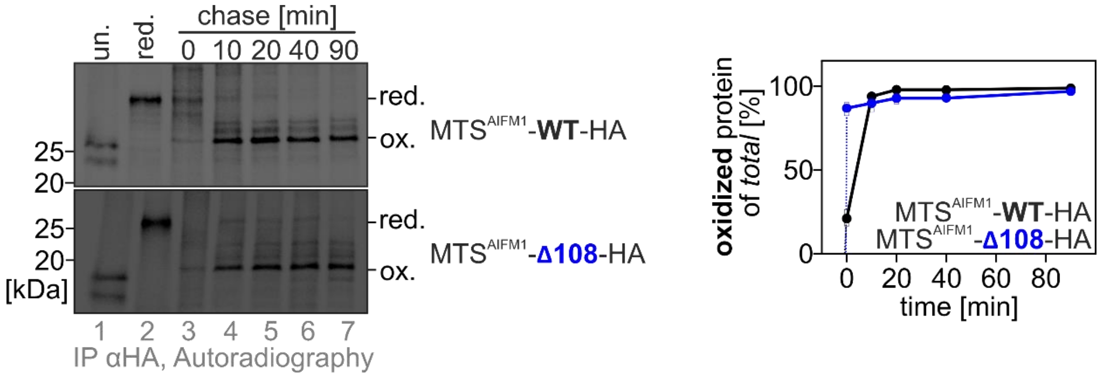

ADDITIONAL FILE 4: Figure S4. In intact cells, MTS^{AIFM1}-MIA40 variants are rapidly oxidized.

Control *in cellulo* oxidation assay to follow oxidative folding of MTS^{AIFM1}-MIA40 variants. Performed as described in **Figure 3E.** Oxidation of MTS^{AIFM1} variants proceeds rapidly indicating rerouting of the IMS import pathway. Quantification using ImageQuantTL. Data from 2 experiments were combined.
