# Supplementary material for: The C-terminal region of the oxidoreductase MIA40 stabilizes its cytosolic precursor during mitochondrial import
Source: BMC Biol. 2020 Aug 6;18:96. doi: 10.1186/s12915-020-00824-1 (PMC7412830; doi:10.1186/s12915-020-00824-1)
Supplement: Supplementary file 5 — Additional file 5: Tables S1-S3. Table S1. Reagents and Ressources, Table S2. Cell lines, and Table S3. Oligonucleotides. [file 12915_2020_824_MOESM5_ESM.pdf]

ADDITIONAL FILE 5: Tabes S1-S3

**Table S1**		
**Reagent/Ressource**	**Source**	**Identifier**
**Antibodies**		
**Mouse monoclonal anti-Strep (1:1000)**	Abcam	Cat# ab184224
**Rabbit polyclonal anti-HA (1:1000)**	Sigma-Aldrich	Cat# SAB4300603; RRID:AB_10620829
**Rat monoclonal anti-HA (3F10; used for immunofluorescence)**	Roche	Cat#11867423001; RRID:AB_10094468
**Mouse monoclonal anti-HA, agarose conjugate**	Sigma-Aldrich	Cat#A2095<br>RRID: AB_257974
**Mouse monoclonal anti-LDH (H-10) (1:1000)**	Santa-Cruz	Cat# sc-133123; RRID:AB_2134964
**Mouse monoclonal anti-PDH (1:1000)**	Santa-Cruz	Cat# sc-377092
**Rabbit polyclonal anti-COX17 (1:1000)**	Biorbyt	Cat# orb160552
**Rabbit polyclonal anti-COX6B1 (1:1000)**	Sigma-Aldrich	Cat# HPA004192, RRID:AB_1847180
**Rabbit polyclonal anti-NDUFB7 (1:1000)**	Abcam	Cat# ab188575
**Rabbit polyclonal anti-NDUFS5 (1:1000)**	Abcam	Cat# ab179806
**Rabbit polyclonal anti-NDUFA8 (1:1000)**	Abcam	Cat# ab184952
**Rabbit polyclonal anti-NDUFB10 (1:1000)**	Abcam	Cat# ab196019
**Rabbit polyclonal anti-CMC1 (1:1000)**	Sigma-Aldrich	Cat#HPA043333; RRID:AB_10797139
**Mouse monoclonal anti-HSP70 (1:1000)**	Santa-Cruz	Cat# sc-66048; RRID:AB_832518
**Rabbit polyclonal anti-TIMM9 (1:250)**	This study	N/A
**Rabbit polyclonal anti-TIMM13 (1:1000)**	Sigma-Aldrich	Cat# HPA048985
**Purified mouse anti-TIMM23 (1:1000)**	BD Biosciences	Cat# 611223
**Rabbit polyclonal anti-STARD7 (1:1000)**	Proteintech	Cat# 15689-1-AP
**Rabbit polyclonal anti-MIA40 (1:1000)**	Fischer et al., 2013,<br>Erdogan et al., 2018	N/A
**Goat anti-Mouse - Affinity Pure, HRP Conjugate**	ImmunoReagents	Cat# GtxMu-003-DHRPX
**Goat anti-Rabbit - Affinity Pure, HRP Conjugate**	ImmunoReagents	Cat# GtxRb-003-DHRPX
**Goat anti-Rat, Alexa Fluor 488**	Invitrogen	Cat# A-11006; RRID:AB_2534074
**Mitotracker red**	Thermo Fisher	Cat# M22425
**Bacterial and Virus Strains**		
**One Shot™ TOP10 Chemically Competent *E. coli***	Thermo Fisher	Cat# C404010
**Rosetta™ 2 (DE3) Singles™ Competent Cells**	Novagen	Cat# 70954-3
**Chemicals**		
**MG132 (Z-Leu-Leu-Leu-al)**	Sigma-Aldrich	Cat# C2211; CAS: 133407-82-6
**Emetine**	Sigma-Aldrich	Cat# E2375; CAS: 7083-71-8
**Methyl-PEG-Maleimide, mmPEG12**	Thermo Fisher	Cat# 22711
**Methyl-PEG-Maleimide, mmPEG24**	Thermo Fisher	Cat# 22713
**N-Ethylmaleimide (NEM)**	Sigma-Aldrich	Ca# E3876; CAS: 128-53-0
**Tris(2-carboxyethyl)phosphine hydrochloride (Tris)**	Sigma-Aldrich	Cat# C4706; CAS: 51805-45-9
**Doxycycline hyclate**	AppliChem	Cat# A2951; CAS: 24390-14-5
**4-Isopropylbenzoic acid (Cumate)**	Sigma-Aldrich	Cat# 268402; CAS: 536-66-3
**Puromycin**	InvivoGen	Cat# ant-pr-1
**Hygromycin B solution from *Streptomyces hygroscopicus***	Sigma-Aldrich	Cat# H0654; CAS: 31282-04-9
**Blasticidin**	InvivoGen	Cat# ant-bl-05
**PrestoBlue™ Cell Viability Reagent**	Invitrogen by Thermo Fisher	Cat# A13261
**Digitonin**	PanReac AppliChem	Cat# A1905
**Benzonase® Nuclease**	Sigma-Aldrich	Cat# E1014
**Critical Commercial Assays**		
**EasyTag EXPRESS 35S Protein Labeling Mix**	Perkin-Elmer	Cat# NEG772
**TnT Quick Coupled Transcription/Translation System**	Promega	Cat# L1170
**GSTrap 4B column**	GE Healthcare	Cat# 28401745
**Superdex 16/600 75 pg High Load column**	GE Healthcare	Cat# 28989333
**FuGENE® HD Transfection Reagent**	Promega	Cat# E2311
**Experimental Models: Cell Lines**		
**Human: Flp-In T-REx-293-cell lines – see Table S2**	Invitrogen	Cat# R78007
**Human: HEK293T-MIA40 CRISPR #2 MIA40 – see Table S2**	Habich et al., 2019	Habich et al., 2019
**Human: Flp-In T-REx-293 CRISPR ko YME1L1 – see Table S2**	Langer lab	

**Oligonucleotides**		
**See Table S3**		
**Recombinant DNA**		
**pcDNA5/FRT/TO Mammalian Expression Vector**	Invitrogen	Fischer et al., 2013
**pOG44**	Invitrogen	Fischer et al., 2013
**PB-CuO-MCS-IRES-GFP-EF1α-CymR-Puro cDNA Inducible Cloning and Expression Vector**	System Biosciences	Cat# PBQM812A-1
**Super PiggyBac Transposase Expression Vector**	System Biosciences	Cat# PB210PA-1
**pGEX-6p-1**	GE Healthcare	Cat# 28954648
**Software and Algorithms**		
**Fiji (*Fiji Is Just ImageJ* 1.51n)**	Schindelin et al., 2012<br>Schneider et al., 2012	imagej.net/Fiji
**Jalview 2.11.0**	Waterhouse et al., 2009	jalview.org
**PyMOL 2.3.4**	The PyMOL Molecular Graphics System, Version 1.2r3pr, Schrödinger, LLC	pymol.org
**Image Lab 5.2**	Biorad Laboratories	
**ImageQuantTL 8.1**	GE Healthcare Life Sciences	
**Clustal Omega**	Sievers and Higgins, 2018<br>Madeira et al., 2019	
**RCSB PDB**	Berman et al., 2000<br>Berman et al., 2003	rcsb.org, wwpdb.org
**JPred4**	Drozdetskiy et al., 2015	www.compbio.dundee.ac.uk/jpred/

**Table S2**				
**Cell lines**	**Plasmid**	**Gene**	**TAG**	**Reference**
**Flp-In T-REx-293-Mock**	pcDNA5/FRT/TO	-	-	Fischer et al., 2013
**Flp-In T-REx-293-MIA40 WT**	pcDNA5/FRT/TO	ORF *MIA40*	Strep (C-terminal)	Fischer et al., 2013
**Flp-In T-REx-293-MIA40 Δ131**	pcDNA5/FRT/TO	ORF *MIA40 Δ131-142*	Strep (C-terminal)	this study
**Flp-In T-REx-293-MIA40 Δ122**	pcDNA5/FRT/TO	ORF *MIA40 Δ122-142*	Strep (C-terminal)	this study
**Flp-In T-REx-293-MIA40 Δ108**	pcDNA5/FRT/TO	ORF *MIA40 Δ108-142*	Strep (C-terminal)	this study
**Flp-In T-REx-293-MIA40 WT**	pcDNA5/FRT/TO	ORF *MIA40*	Strep (N-terminal)	this study
**Flp-In T-REx-293-MIA40 Δ108**	pcDNA5/FRT/TO	ORF *MIA40 Δ108-142*	Strep (N-terminal)	this study
**Flp-In T-REx-293-MIA40 WT**	pcDNA5/FRT/TO	ORF *MIA40*	-	this study
**Flp-In T-REx-293-MIA40 Δ131**	pcDNA5/FRT/TO	ORF *MIA40 Δ131-142*	-	this study
**Flp-In T-REx-293-MIA40 Δ122**	pcDNA5/FRT/TO	ORF *MIA40 Δ122-142*	-	this study
**Flp-In T-REx-293-MIA40 Δ108**	pcDNA5/FRT/TO	ORF *MIA40 Δ108-142*	-	this study
**Flp-In T-REx-293-MIA40 WT**	pcDNA5/FRT/TO	ORF *MIA40*	HA (C-terminal)	this study
**Flp-In T-REx-293-MIA40 Δ131**	pcDNA5/FRT/TO	ORF *MIA40 Δ131-142*	HA (C-terminal)	this study
**Flp-In T-REx-293-MIA40 Δ122**	pcDNA5/FRT/TO	ORF *MIA40 Δ122-142*	HA (C-terminal)	this study
**Flp-In T-REx-293-MIA40 Δ108**	pcDNA5/FRT/TO	ORF *MIA40 Δ108-142*	HA (C-terminal)	this study
**Flp-In T-REx-293-MIA40 WT**	pcDNA5/FRT/TO	ORF *MIA40*	HA (N-terminal)	this study
**Flp-In T-REx-293-MIA40 Δ108**	pcDNA5/FRT/TO	ORF *MIA40 Δ108-142*	HA (N-terminal)	this study
**Flp-In T-REx-293-MIA40 Δ108**	pcDNA5/FRT/TO	ORF *MIA40 Δ1-40*	HA (C-terminal)	this study
**Flp-In T-REx-293-MIA40 Δ108 + 14 positively charged aa**	pcDNA5/FRT/TO	ORF *MIA40 Δ108-142 + 14 positively charged aa*	-	this study
**Flp-In T-REx-293-MIA40 Δ108 + 14 neutral charged aa**	pcDNA5/FRT/TO	ORF *MIA40 Δ108-142 + 14 positively charged aa*	-	this study
**Flp-In T-REx-293-MIA40 Δ108 + GSS**	pcDNA5/FRT/TO	ORF *MIA40 Δ108-142 + GSS*	-	this study
**Flp-In T-REx-293-MIA40 Δ131 + 12 positively charged aa**	pcDNA5/FRT/TO	ORF *MIA40 Δ131-142 + 12 neutral charged aa*	-	this study
**Flp-In T-REx-293-MIA40 Δ131 + 12 neutral charged aa**	pcDNA5/FRT/TO	ORF *MIA40 Δ131-142 + 12 neutral charged aa*	-	this study
**Flp-In T-REx-293-MIA40 Δ131 + GSS**	pcDNA5/FRT/TO	ORF *MIA40 Δ131-142 + GSS*	-	this study
**Flp-In T-REx-293-COX19**	pcDNA5/FRT/TO	ORF *COX19*	HA (C-terminal)	Fischer et al., 2013
**Flp-In T-REx-293-COX19-C-MIA**	pcDNA5/FRT/TO	ORF *COX19 + aa108-142 MIA40*	HA (C-terminal)	this study
**Flp-In T-REx-293-COX19-C-neutral**	pcDNA5/FRT/TO	ORF *COX19 + 35 neutral charged aa*	HA (C-terminal)	this study
**Flp-In T-REx-293-COX19 4CS**	pcDNA5/FRT/TO	ORF *COX19* (G89,119, 152, 182►C)	HA (C-terminal)	Habich et al., 2019
**Flp-In T-REx-293-COX19 4CS-C-MIA**	pcDNA5/FRT/TO	ORF *COX19* (G89,119, 152, 182►C) + *aa108-142 MIA40*	HA (C-terminal)	this study
**Flp-In T-REx-293-COX19 4CS-C-neutral**	pcDNA5/FRT/TO	ORF *COX19* (G89,119, 152, 182►C) + *35 neutral charged aa*	HA (C-terminal)	this study

**Flp-In T-REx-293-AIF^{MTS}-MIA40 WT**	pcDNA5/FRT/TO	ORF *AIF^{MTS}-MIA40*	-	this study
**Flp-In T-REx-293-AIF^{MTS}-MIA40 Δ108**	pcDNA5/FRT/TO	ORF *AIF^{MTS}-MIA40 Δ108-142*	-	this study
**Flp-In T-REx-293-SOD2**	pcDNA5/FRT/TO	ORF *SOD2*	HA (C-terminal)	this study
**HEK293T**	-	-	-	Habich et al., 2019
**HEK293T MIA40 knockout #2-Mock**	PB-CuO-MCS- IRES-GFP-EF1α-CymR-Puro	-	-	Habich et al., 2019
**HEK293T MIA40 knockout #2-MIA40 WT**	PB-CuO-MCS- IRES-GFP-EF1α-CymR-Puro	ORF *MIA40*	-	Habich et al., 2019
**HEK293T MIA40 knockout #2-C53S**	PB-CuO-MCS- IRES-GFP-EF1α-CymR-Puro	ORF *MIA40* (T157►G)	-	Habich et al., 2019
**HEK293T MIA40 knockout #2-MIA40 Δ131**	PB-CuO-MCS- IRES-GFP-EF1α-CymR-Puro	ORF *MIA40 Δ131-142*	-	This study
**HEK293T MIA40 knockout #2-MIA40 Δ122**	PB-CuO-MCS- IRES-GFP-EF1α-CymR-Puro	ORF *MIA40 Δ131-122*	-	This study
**HEK293T MIA40 knockout #2-MIA40 Δ108**	PB-CuO-MCS- IRES-GFP-EF1α-CymR-Puro	ORF *MIA40 Δ131-108*	-	This study
**Flp-In T-REx-293-YME1L1 ko**	-	-	-	Hartmann et al, 2016
**Flp-In T-REx-293-YME1L1 ko-Mock**	PB-CuO-MCS- IRES-GFP-EF1α-CymR-Puro	-	-	This study
**Flp-In T-REx-293-YME1L1 ko-MIA40 WT**	PB-CuO-MCS- IRES-GFP-EF1α-CymR-Puro MIA40 WR	ORF *MIA40 WT*	-	This study
**Flp-In T-REx-293-YME1L1 ko-MIA40 Δ108**	PB-CuO-MCS- IRES-GFP-EF1α-CymR-Puro MIA40 Δ108	ORF *MIA40 Δ108-142*	-	This study
**Flp-In T-REx-293-YME1L1 ko + YME1L1**	-	-	-	Hartmann et al, 2016
**Flp-In T-REx-293-YME1L1 ko + YME1L1**	PB-CuO-MCS- IRES-GFP-EF1α-CymR-Puro	-	-	This study
**Flp-In T-REx-293-YME1L1 ko + YME1L1-MIA40**	PB-CuO-MCS- IRES-GFP-EF1α-CymR-Puro MIA40 WR	ORF *MIA40 WT*	-	This study
**Flp-In T-REx-293-YME1L1 ko + YME1L1-MIA40 Δ108**	PB-CuO-MCS- IRES-GFP-EF1α-CymR-Puro MIA40 Δ108	ORF *MIA40 Δ108-142*	-	This study
**Flp-In T-REx-293**	-	-	-	Thermo Fisher Scientific
**Flp-In T-REx-293 -Mock**	PB-CuO-MCS- IRES-GFP-EF1α-CymR-Puro	-	-	This study
**Flp-In T-REx-293 -MIA40 WT**	PB-CuO-MCS- IRES-GFP-EF1α-CymR-Puro MIA40 WR	ORF *MIA40 WT*	-	This study
**Flp-In T-REx-293 -MIA40 Δ108**	PB-CuO-MCS- IRES-GFP-EF1α-CymR-Puro MIA40 Δ108	ORF *MIA40 Δ108-142*	-	This study
**Rosetta2 (DE3)-MIA40 WT**	pGEX-6p-1	ORF *MIA40*	GST (N-terminal)	Friederich et al., 2017
**Rosetta2 (DE3)-PreScission Protease**	pGEX-6p-1	ORF PreScission Protease	GST (N-terminal)	Friederich et al., 2017
**Rosetta2 (DE3)-MIA40 Δ131**	pGEX-6p-1	ORF *MIA40 Δ131-142*	GST (N-terminal)	this study
**Rosetta2 (DE3)-MIA40 Δ122**	pGEX-6p-1	ORF *MIA40 Δ122-142*	GST (N-terminal)	this study
**Rosetta2 (DE3)-MIA40 Δ108**	pGEX-6p-1	ORF *MIA40 Δ108-142*	GST (N-terminal)	this study
**Rosetta2 (DE3)-MIA40 WT**	pRSET-A	ORF *MIA40*	6xHis (N-terminal)	this study
**Rosetta2 (DE3)-MIA40 Δ108**	pRSET-A	ORF *MIA40 Δ108-142*	6xHis (N-terminal)	this study

Table S3

Oligonucleotides	Sequence (5‘-3‘)	Restriction site
**Fw for pcDNA5/FRT/TO MIA40 WT, MIA40 Δ131, MIA40 Δ122, MIA40 Δ108, MIA40 Δ131-Strep, MIA40 Δ122-Strep, MIA40 Δ108-Strep, MIA40-HA, MIA40 Δ131-HA, MIA40 Δ122-HA, MIA40 Δ108-HA, MIA40 108+14 pos. aa, MIA40 108+14 neut. aa, MIA40 108+GSS, MIA40 131+12 pos. aa, MIA40 131+12 neut. aa, MIA40 131+GSS**	GGCGGATCCGCAGCCATGTCCTATTGCCG	BamHI
**Rev for pcDNA5/FRT/TO MIA40 Δ131-Strep**	CTACTCGAGTTATTTCTCAAATTGTGGATGACTCCATC CTCCAGCAATGGGAGCTGTTTCTTCTG	XhoI
**Rev for pcDNA5/FRT/TO MIA40 Δ122-Strep**	CTACTCGAGTTATTTCTCAAATTGTGGATGACTCCATCCTCCAG CTGCTGGCTTCTTCTCTCTTTCC	XhoI
**Rev for pcDNA5/FRT/TO MIA40 Δ108-Strep**	CAACTCGAGTTATTTCTCAAATTGTGGATGACTCCATCCTCCAG CTTGGGGATAGAGGTCTGGGTATTTCTGC	XhoI
**Fw for pcDNA5/FRT/TO Strep-MIA40 WT, Strep-MIA40 Δ108**	ATTGGATCCGCAGCCATGTGGAGTCATCCACAATTTGAGAAAG GAGCTTCCTATTGCCGGCAGG	BamHI
**Rev for pcDNA5/FRT/TO MIA40 WT, Strep-MIA40 WT, HA-MIA40 WT, AIF^{MTS}-MIA40 WT**	CGGCTCGAGTTAACTTGATCCCTCCTCTTCTTTGGTTGCAGTGG	XhoI
**Rev for pcDNA5/FRT/TO MIA40 Δ108, Strep-MIA40 Δ108, HA-MIA40 Δ108, AIF^{MTS}-MIA40 Δ108**	CGGCTCGAGTTATTGGGGATAGAGGTCTGGGTATTTCTGCATG CATTC	XhoI
**Rev for pcDNA5/FRT/TO MIA40 Δ131**	CGGCTCGAGTTAAATGGGAGCTGTTTCTTCTGCTTGTTCTGC	XhoI
**Rev for pcDNA5/FRT/TO MIA40 Δ122**	CGGCTCGAGTTATGCTGGCTTCTTCTCTCTTTCCTCTTCC	XhoI
**Rev for pcDNA5/FRT/TO MIA40 WT-HA, MIA40 Δ40-HA**	GTACTCGAGTTAAGCATAATCTGGAACATCATATGGATATCCA GCACTTGATCCCTCCTCTTC	XhoI
**Fw for pcDNA5/FRT/TO MIA40 Δ40-HA**	GATGGATCCGCAGCCATGGGATTGATACTGCCAAATGG	BamHI
**Rev for pcDNA5/FRT/TO MIA40 Δ131-HA**	GTACTCGAGTTAAGCATAATCTGGAACATCATATGGATATCCA GCAATGGGAGCTGTTTCTTCTGC	XhoI
**Rev for pcDNA5/FRT/TO MIA40 Δ122-HA**	GTACTCGAGTTAAGCATAATCTGGAACATCATATGGATATCCA GCTGCTGGCTTCTTCTCTCTTTCC	XhoI
**Rev for pcDNA5/FRT/TO MIA40 Δ108-HA**	GTACTCGAGTTAAGCATAATCTGGAACATCATATGGATATCCA GCTTGGGGATAGAGGTCTGG	XhoI
**Fw pcDNA5/FRT/TO HA-MIA40 WT, HA-MIA40 Δ108**	ATTGGATCCGCAGCCATGTATCCATATGATGTTCCAGATTATG CTGGAGCTTCCTATTGCCGGCAGGAAGG	BamHI
**Rev for pcDNA5/FRT/TO MIA40 Δ108+14 pos. aa**	CTACTCGAGTTATGCTGGCTTCTTTCTTCTCTTCCTCTTCCTCTT TCTCTTCCTTTGGGGATAGAGGTCTGGG	XhoI
**Rev for pcDNA5/FRT/TO MIA40 Δ108+14 neut. aa**	ATACTCGAGTTATGCTGGTGAACCTGCAGCCCCTGAACCAGAA GCTCCAGAAGCTTGGGGATAGAGGTCTGGG	XhoI
**Rev for pcDNA5/FRT/TO MIA40 Δ108+GSS**	CGACTCGAGTTAACTTGATCCTTGGGGATAGAGGTCTGGGTA	XhoI
**Rev for pcDNA5/FRT/TO MIA40 Δ131+12 pos. aa**	GTACTCGAGTTAACTTGATCCACGCTTACGCTTAGTAGCTGTTG CCCGAATGGGAGCTGTTTCTTCTG	XhoI
**Rev for pcDNA5/FRT/TO MIA40 Δ131+12 neut. aa**	GTACTCGAGTTAACTTGATCCTGCAGCAGCAGTAGTAGCTGTT GCAGCAATGGGAGCTGTTTCTTCTG	XhoI
**Rev for pcDNA5/FRT/TO MIA40 Δ131+GSS**	GTACTCGAGTTAACTTGATCCAATGGGAGCTGTTTCTTCTGC	XhoI
**Fw (1) for pcDNA5/FRT/TO COX19-C-MIA, pcDNA5/FRT/TO COX19-4SC-C-MIA, pcDNA5/FRT/TO COX19-C-neutral, pcDNA5/FRT/TO COX19-4SC-C-neutral**	TATGGATCCGCCAACATGTCGACCGCCATGAATTTC	BamHI
**Fw (2) for pcDNA5/FRT/TO COX19-C-MIA, pcDNA5/FRT/TO COX19-4SC-C-MIA**	GGAAAATCAGAGGCAAAAAAAGAGGATGAGGATGAGG	-
**Rev (1) for pcDNA5/FRT/TO COX19-C-MIA, pcDNA5/FRT/TO COX19-4SC-C-MIA**	CCTCATCCTCATCCTCTTTTTTTGCCTCTGATTTTCC	-
**Rev (2) for pcDNA5/FRT/TO COX19-C-MIA, pcDNA5/FRT/TO COX19-4SC-C-MIA**	GTACTCGAGTTAAGCATAATCTGGAACATCATATGGATATCCA GCACTTGATCCCTCCTCTTC	XhoI
**Fw (2) for pcDNA5/FRT/TO COX19-C-neutral,**	GGAAAATCAGAGGCAAAAAAAGCTTCTGGAGCTTCTGGTTC	-
